# Effect of hydrogen peroxide and carbon-to-nitrogen ratio on growth and biochemical profile in oleaginous mucoromycota

**DOI:** 10.1186/s12934-025-02863-1

**Published:** 2025-11-12

**Authors:** Cristian Bolaño-Losada, Boris Zimmermann, Svein Jarle Horn, Achim Kohler, Volha Shapaval

**Affiliations:** 1 Faculty of Science and Technology, Norwegian University of Life Sciences (NMBU), Ås, 1432 Norway; 2 Faculty of Chemistry, Biotechnology and Food Science, Norwegian University of Life Sciences (NMBU), Ås, 1432 Norway

**Keywords:** Mucoromycota, Hydrogen peroxide, Carbon-to-nitrogen ratio, Redox biology, Vibrational spectroscopy.

## Abstract

**Background:**

Hydrogen peroxide (H_2_O_2_) has gained attention as cofactor of lytic polysaccharide monooxygenases (LPMOs) during lignocellulose saccharification. The action of these enzymes has been shown to significantly enhance saccharification efficiency. However, in simultaneous saccharification and fermentation (SSF) processes, H_2_O_2_ can have deleterious effects on the fermenting microorganism. In addition to oxidative stress, at certain concentration ranges, H_2_O_2_ can play a crucial role in redox biology mediating metabolic crosstalk. Indeed, some works have explored the influence of H_2_O_2_ and other stress molecules in lipid accumulation. In this study, nine strains from eight different species of Mucoromycota were grown at different sublethal concentrations of H_2_O_2_ and two carbon-nitrogen (C/N) ratios. The aim of this study was to investigate whether H_2_O_2_ could enhance lignocellulose-based SSF with oleaginous Mucoromycota fungi to produce second-generation biofuels. Therefore, effects of H_2_O_2_ concentration, beneficial or deleterious, were identified under different C/N conditions.

**Results:**

In general, all the strains tolerated H_2_O_2_ at much higher concentrations than those commonly used to improve enzymatic saccharification (1-19 mM vs 1-240 µM). Vibrational spectroscopy (mid-infrared and Raman) was used to analyze the biochemical composition of the fungi. The exposure to sublethal H_2_O_2_ doses did not increase any metabolite in particular but slightly reduced biomass production at concentrations near the minimal inhibitory concentration (MIC) in some cases. For *Lichtheimia corymbifera* grown in standard C/N medium, an accumulation of intracellular proteins with oxidative damage was positively correlated to the H_2_O_2_ concentration. This was not observed for other strains. The biggest changes in the biochemical composition of the fungal biomass were linked to changes in medium C/N ratios. This included different carbon allocation strategies among the tested species, such as accumulation of lipids and polyphosphates, lipids and saccharides, etc.

**Conclusions:**

Our results suggest that the Mucoromycota strains used in this study are compatible with H_2_O_2_ feeding in lignocellulose-based SSF to enhance efficiency while sustaining minimal risk of oxidative damage.

**Graphical abstract:**

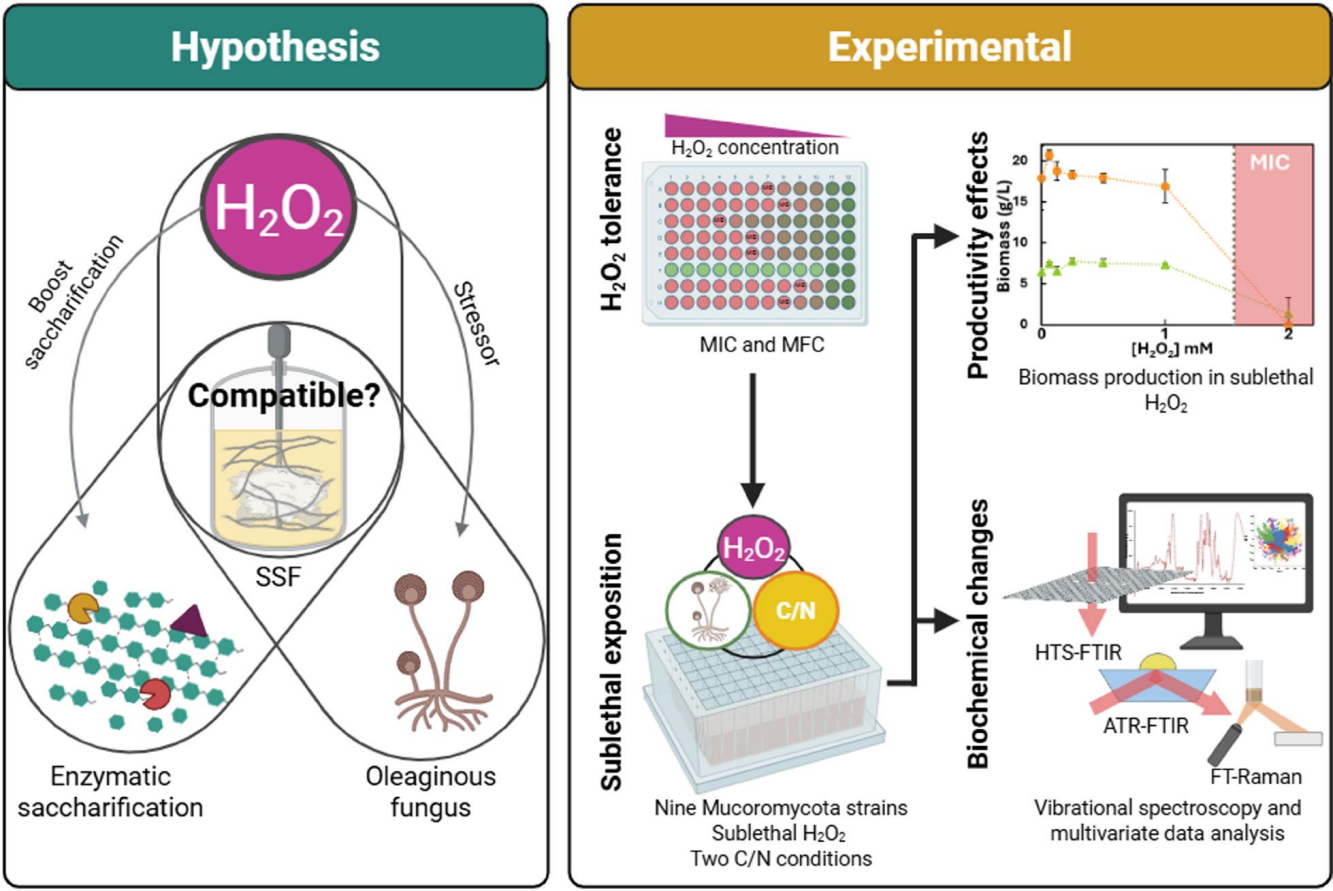

**Supplementary Information:**

The online version contains supplementary material available at 10.1186/s12934-025-02863-1.

## Background

Mucoromycota is a phylum of filamentous fungi represented by many species possessing oleaginicity, which is the ability to accumulate lipids in amounts above 20% over their dry weight [1, 2]. Some species can even reach the astonishing amount of 80% [3]. Lipogenesis in Mucoromycota fungi has been extensively studied using omics technologies [4–6]. The most effective and studied strategy for inducing lipid accumulation in oleaginous Mucoromycota is cultivation in high carbon-to-nitrogen (C/N) ratio media, where nitrogen is limited and carbon is in excess [7]. Growth in high C/N and nitrogen-limited media occurs in two distinct phases. The first phase, the trophophase, is characterized by active cell proliferation driven by available nitrogen, which is essential for protein and nucleic acid synthesis. The second phase, the idiophase, begins when nitrogen limitation halts cell proliferation, while carbon remains accessible and its assimilation by the cells leads to lipid accumulation [2, 8]. During the idiophase, a metabolic reorganization occurs, leading to the overproduction of acetyl-CoA and NADPH. These molecules serve, respectively, as building block and the reducing power necessary for the biochemical synthesis of acetyl glycerides.

In our previous study we explored compatibility of oleaginous Mucoromycota with simultaneous saccharification and fermentation (SSF) [9]. The interest in this process is driven by the potential to produce second-generation biodiesel from lignocellulose, a renewable and non-food feedstock, while reducing process time and costs. Over the past decade, the significant role of accessory enzymes known as lytic polysaccharide monooxygenases (LPMOs) has been highlighted, particularly for their ability to disrupt the crystallinity of cellulose [10]. LPMOs can use H_2_O_2_ as a cofactor; when H_2_O_2_ is added to a cellulose enzymatic saccharification with a cellulase cocktail containing LPMO, the hydrolysis efficiency is enhanced [11]. Recent studies have explored the use of H_2_O_2_ to improve hydrolysis efficiency to reduce operational costs, with 30–39% increases at flask [12] and 33% increases at bioreactor pilot scale [13] under specific conditions. Nonetheless, in fermentation strategies such as SSF, feeding with H_2_O_2_ can be disruptive for microorganism growth. For instance, some studies reported oxidative damage and yield reduction when excessive H_2_O_2_ concentrations were applied in a cellulose-based SSF for lactic acid production with bacteria [14].

On the other hand, although reactive oxygen species (ROS) are primarily associated with oxidative stress, some studies have highlighted their involvement in certain metabolic pathways by redox signaling [15]. Hypothetically, ROS species (H_2_O_2_, O_2_⁻, and •OH) can act as Janus-faced molecules, exhibiting both beneficial and detrimental effects depending on their concentration. Redox signaling inside the cells is estimated to occur at the nanomolar levels, while oxidative damage arises at higher concentrations [16]. Not all ROS species are equally involved in redox signaling, with key differences between H_2_O_2_, O_2_⁻, and •OH. These signaling mechanisms depend on changes in protein conformation triggered by the oxidation of specific cysteine residues [17]. In particular, the oxidation of cysteine residues by H₂O₂, results in the formation of sulfenic acid (Cys-SOH) and the induction of allosteric changes in the proteins that are reversible, unlike O_2_⁻ and •OH. H_2_O_2_ is produced by NADPH oxidases and superoxide dismutase (SOD) enzymes, both of which are linked to stress-sensing systems (e.g., Ca²⁺ sensors) in the cell. This highlights the role of H_2_O_2_ in redox signaling.

Notably, the induction of lipogenesis with ROS, such as H_2_O_2_, has been reported in various microorganisms, including yeast [18], algae [19–21], and even rat liver cells [22]. This emerging link underscores the importance of further understanding the regulation of lipogenesis, particularly for selecting cultivation parameters that enhance the efficient production of single-cell oils (SCO). However, although a proteomic study of *Mucor circinelloides* revealed that redox homeostasis proteins are upregulated during lipogenesis triggered by nitrogen deficiency [6], it remains unknown whether lipogenesis can be induced by H_2_O_2_ exposure in filamentous fungi.

Therefore, understanding how Mucoromycota species respond to H_2_O_2_ is essential to anticipate potential oxidative damage or redox induced of specific metabolites accumulation when applied in SSF strategies. In this study, the effects of H_2_O_2_ in a set of Mucoromycota strains were studied because two reasons: (1) to determine tolerance towards H_2_O_2_ for their compatibility in SSF with H_2_O_2_-enhanced saccharification in line with our previous work [9], and (2) to determine whether sublethal concentrations promote lipogenesis or deleterious effects. The effects of H_2_O_2_ were studied under two C/N medium conditions, one with balanced carbon and nitrogen (standard C/N) and the other with an excess of carbon over nitrogen (high C/N).

The H_2_O_2_ tolerance of nine Mucoromycota fungi strains was determined and subsequentially grow at various C/N and sublethal H_2_O_2_ conditions. Vibrational (infrared and Raman) spectroscopy and Duetz microtiter plate cultivation system were combined to establish a high-throughput setup capable of screening the full range of tested conditions. Vibrational spectroscopy has proven to be a fast and reliable technique for comprehensive assessment of biochemical composition changes in microbial biomass [8, 23–25]. Meanwhile, the Duetz system offers an excellent platform for microorganism cultivation with optimal aeration, reproducibility and scalability with reduced cross-contamination and evaporation [26, 27].

## Materials and methods

### Fungal strains

Nine Mucoromycota strains belonging to eight species and seven genera were used in this study. The strains were obtained in lyophilized form from the Czech Collection of Microorganisms (CCM; Brno, Czech Republic), the Norwegian School of Veterinary Science (VI; Ås, Norway), the All-Russian Collection (VKM, Pushchino, Russia), and the American Type Culture Collection (ATCC; Manassas, United States). The strain selection was based on lipid accumulation capacity, diversity of fatty acid profiles, SSF potential, and available background information from previous studies [1, 3, 9, 28, 29]. The list of strains is presented in Table 1.

**Table 1:** List of the fungal strains used in the study with their respective collection number and abbreviations

Species	Collection number	Abbreviation
*Lichtheimia corymbifera*	CCM F8077	LC
*Absidia glauca*	CCM F451	AG
*Cunninghamella blakesleeana*	CCM F705	CB
*Mortierella alpina*	ATCC 32222	MA
*Mucor circinelloides*	CCM F220	MC
*Mucor circinelloides*	VI04473	MCVI
*Mortierella hyalina*	VKM F1629	MH
*Rhizopus stolonifer*	VKM F400	RS
*Umbelopsis vinacea*	CCM F539	UV

### Storage of stock cultures and Preparation of spore inoculum

All the strains were cultivated in malt extract agar (MEA) (Merck, Germany) at 25 °C for 7–14 days until a good level of sporulation was obtained. MEA plates were prepared by dissolving 50 g of MEA powder in 1 L of distilled water and autoclaved at 121 °C for 15 min. The spores were collected by adding 10 mL of sterile 0.9% NaCl solution to the plates and dragging the mycelium with a flame-sterilized glass Digralsky spreader. The spore suspensions were mixed with 33%_v/v_ glycerol and stored in cryovials at – 80 °C, except for *Mortierella alpina*, which was sub-cultured regularly by transferring agar plugs with mycelium to fresh MEA media.

### Chemicals

All the chemicals used in this study were purchased from Merck (Germany).

### H_2_O_2_ minimal inhibitory concentration (MIC) and minimal fungicidal concentration (MFC)

The tolerance to H_2_O_2_ for all selected fungal strains, except *M. alpina*, was assessed by determining the minimal inhibitory concentration (MIC) using the microtiter broth dilution method in 96-well microplates. The spore solution was obtained from MEA cultivation as described above. The spore solution was clarified through two layers of sterile Miracloth (22–25 μm pore size, Merck, Germany) to remove mycelial fragments. The spores were counted using an automated cell counter Countess 3 (Invitrogen, United States). Malt extract broth (MEB) (Merck, Germany) with composition (g/L): malt extract, 17; mycological peptone, 3; and pH 5.4; was used as the growth medium. Two 2-fold concentrated MEB solutions with different H_2_O_2_ concentrations (200 µM and 150 µM) were prepared, followed by nine 2-fold serial dilutions in 2-fold concentrated MEB in a sterile flat-bottom polystyrene 96-well plate. 100 µL of spore inoculum with spore concentration 4 × 10^4^ spores/mL of each strain was added to each well containing 100 µL of 2-fold concentrated MEB media with the corresponding H_2_O_2_ concentrations or without H_2_O_2_ (control). The treatments were prepared duplicated for each H_2_O_2_ concentration and each fungal strain. The microplates were covered by a sterile vented polystyrene lid, placed inside zip bags semi-open to avoid evaporation, and incubated in a static incubator at 25 °C for 48 h. After the incubation, the optical density was recorded at 600 nm with a UV/Vis spectrophotometer SPECTROstar Nano (BMG Labtech, Germany). MIC was defined as the lowest H_2_O_2_ concentration that inhibited growth. Further, the minimal fungicidal concentration (MFC) was also determined by plating 100 µL of the wells showing no fungal growth on MEA. After incubation at 25 °C for 48 h, the colonies were counted. MFC was defined as the minimal concentration of H_2_O_2_ that reduced the germination of the initial spore concentration by 99.9% (3-log reduction in colony forming units).

In the case of *M. alpina*, the production of spores in laboratory conditions was difficult, which is general for this species [30]. Therefore, to determine the MIC value, *M. alpina* was prepared from an active mycelium solution. The mycelium biomass was prepared by adding two smashed mycelium agar plugs, cut with a cork borer no. 3, into 20 mL of MEB in a 100 mL flask with a cotton plug, and cultivated at 25 °C, 180 rpm for 72 h in a MaxQ™ 4000 shaker (Thermo Scientific, USA). The obtained *M. alpina* biomass was filtered using a Steritop™ filter of 0.22 μm pore size and washed thoroughly with a sterile 0.9% NaCl solution. An aliquot of fresh mycelium was taken to determine the moisture content of the wet biomass in a Kern DBS 60−3 moisture analyzer (Kern & Sohn, Germany). The remaining fresh biomass was transferred into a sterile 50 mL Falcon tube with 0.9% NaCl solution to achieve a concentration of 10 g/L of dry biomass. The solution was homogenized following the recommendations of Zweck et al. [31] with the help of a Fisherbrand 150 homogenizer (Fisher Scientific, US) equipped with an autoclaved stainless-steel 7 mm diameter probe (Fisher Scientific, cat. No. 15595819) for 10–15 s at medium speed. The homogenate was further diluted with 0.9% NaCl to get a solution of 0.2 g/L and used as inoculum. For the MIC determination, a 24-extra high deep well microplate and Duetz microtiter plate system (MTPS) (Enzyscreen, The Netherlands, Cat #CR1424hd) with a sandwich cover of spongy silicone and filters was used [26]. Each well was filled with 3.5 mL of MEB with or without H_2_O_2_ (2-fold concentrated) and 3.5 mL of 0.2 g/L *M. alpina* mycelium suspension. The total volume for each well was 7 mL and 0.1 g/L of dry-weight mycelium. A range with the following H_2_O_2_ concentrations 0.1875, 0.375. 0.5, 0.75, 1, 1.5, 2, 3, 4, 8, 16 mM was used, including a control without H_2_O_2_. Each treatment was performed in duplicate. The well plate was incubated at 25 °C and 400 rpm for 48 h. After the incubation, the fungal biomass was washed with 0.9% NaCl three times by spinning for 10 min at 10000 rpm and discarding the supernatant. The washed biomass was freeze-dried using a Labconco Freezone 2.5 L (Labconco, United States). Thus, MIC corresponded to the minimal concentration that inhibited biomass growth by more than 95% compared to the control.

### Evaluation of the effect of sub-lethal H_2_O_2_ concentration

To assess the impact of H_2_O_2_ on the mycelial composition and its potential to induce lipogenesis at sublethal doses under varying C/N ratios, an analogous method to the microtiter broth dilution assay was employed. Two media with different C/N ratios were used. Generally, the average C/N ratio of fungal biomass ranges from 10 to 20 [32]. Therefore, we considered “standard” (abbreviated as std) a medium with C/N equal to 18.8 that is in the previously presented range. On the other hand, we named “high” a medium with C/N equal to 89, which promotes an unbalanced scenario where nitrogen is limited, and carbon is allocated towards lipid and other metabolite accumulation. The composition of the media was (g/L): glucose, 17 (for standard C/N) and 80 (for high C/N); peptone, 3; Na_2_HPO_4_, 2; KH_2_PO_4_, 7; MgSO_4_.7H_2_O, 1.5; CaCl_2_.2H_2_O, 0.1; FeCl_3_·6H_2_O 0.008; ZnSO_4_·7H_2_O, 0.001; CuSO_4_·5H_2_O, 0.0001; Co(NO_3_)_2_·H_2_O 0.0001; MnSO_4_·5H_2_O, 0.0001 and pH 6.5. An organic nitrogen source was preferred over an inorganic one since its buffering effect makes the pH more stable and avoids the production of some metabolite linked to sharp pH decreases [28, 33]. Peptone was chosen to keep the same nitrogen source as in the MEB used for MIC determination. The total nitrogen and carbon content of peptone (WVR cat. no. J636-500 g, Lot. 23l2556822) was 12% and 40%, respectively, determined by the Dumas method following the ISO 16634 [34]. The carbon content of peptone was not considered for the C/N ratio calculation, if so, values would be 22.2 and 92.2 for standard and high respectively.

Various concentrations of H_2_O_2_ were prepared as 2-fold concentrated solutions in both standard and high C/N media. The sublethal H_2_O_2_ concentrations applied to each strain were selected according to their respective MIC values and can be found in Additional file 1: Table S1. Polypropylene square 96-deep well microtiter plates (MTPs) (Enzyscreen, The Netherlands, Cat #CR1496) were used for the 2-fold serial dilutions, generating different sublethal concentrations of H_2_O_2_. Each well was filled with 400 µL of 2-fold concentrated medium with H_2_O_2_ or without (control), followed by the addition of 400 µL of inoculum to reach a final spore concentration of 2 × 10^4^ spores/mL. In the case of *M. alpina*, the inoculum was prepared from four agar plugs containing fresh live mycelium, cut with a cork borer no. 5 (10 mm Ø), smashed, and resuspended in 10 mL of 0.9% NaCl sterile solution. Each cultivation condition was performed in triplicate.

The microplates were incubated in an orbital shaker at 25 °C and 400 rpm for 5 days. Following incubation, the microplates were centrifuged at 4500 rpm and 4 °C for 15 min in a Hermle Z326K centrifuge (Hermle, Germany). The fermentation supernatants were separated and stored in 1.1 mL deep 96-well plates, while the pellets were washed with distilled water by three cycles of resuspension in water, centrifugation, and supernatant discard. Both supernatant and fungal biomass samples were stored at -20 °C until further analysis.

### Fourier transform infrared high-throughput screening (FTIR-HTS) spectroscopy

Samples exposed to the sublethal concentrations of H_2_O_2_ were processed directly in the same 96-well microplate used for incubation. To each well, 250 mg of acid-washed beads (710–1180 μm diameter, Merck, Germany) were added with the help of a LAbTIE™ bead dispenser (MolGen, The Netherlands), followed by the addition of 300–400 µL of distilled water. The microplate was closed with a 96-well plate square shape silicon mat lid, and the biomass was homogenized by bead beating using a Bead Ruptor 96 disruptor (Omni International, United States), performing 3 cycles of 3.5 min each at 25 Hz with a resting time of 30 s between cycles. The homogenate was used for spectra acquisition by FTIR-HTS.

For the acquisition of FTIR spectra, a high-throughput screening module (HTS-XT) coupled to Vertex70-FTIR spectrometer (both Bruker Optik GmbH, Germany) equipped with a globar mid-IR source and a deuterated L-alanine doped triglycene sulfate (DLaTGS) detector was used. Drops of 8 µL homogenized biomass were dispensed onto an IR-transparent 384-well silicon microplate (Bruker Optik GmbH, Germany) and dried at room temperature. Three replicates from each sample were dispensed on the plate for spectra acquisition. The spectra were acquired in transmission mode, with a total of 64 scans, spectral resolution of 6 cm^− 1^, digital spacing of 1.928 cm^− 1^, over the range of 4000 to 400 cm^− 1^, using Blackman-Harris 3-Term apodization, and an aperture of 6 mm. Spectra were recorded as the ratio of the sample spectrum to the spectrum of the empty IR transparent microplate. The OPUS 8.2 software (Bruker Optik GmbH, Ettlingen, Germany) was used for data acquisition and instrument control. 1162 spectra were acquired from a total of 404 samples, 50 technical replicates of low-quality were not considered for the analysis due to low absorbance derived from reduced amount of biomass.

### FT-Raman spectroscopy

Raman spectra were only acquired from the biomass of the *M. circinelloides* strains (MC and MCVI) grown at standard and high C/N medium. Raman spectra were only acquired from the biomass of the *M. circinelloides* strains (MC and MCVI) since only the biomass of these two strains had significant amount of carotenoid pigments. FT-Raman spectroscopy complements FTIR by enabling the detection of carotenoid pigments through the resonant Raman effect, which enhances the vibrational signals of these molecules. The measurements were done with a MultiRAM FT-Raman spectrometer (Bruker Optik GmbH, Germany) equipped with a neodymium doped ytrrium aluminum garnet (Nd: YAG) laser (1064 nm, 9394 cm^− 1^) and a germanium detector cooled with liquid nitrogen, equipped with a High Throughput Screening (HTS) Mapping Stage with 2.5 mm aperture and a collecting mirror objective. Approximately 10–20 mg of freeze-dried biomass was placed in a 400 µL flat bottom glass insert vial. The sample vials were placed in a 96-well microplate coupled to the HTS stage with the laser focused on the bottom of the vial. The FT-Raman spectra were acquired in backscattering geometry with a total of 2048 scans, using Norton-Beer medium apodization, spectral resolution of 8 cm^− 1^, and digital spacing of 1.928 cm^− 1^, over the range 3785–45 cm^− 1^, at 500 mW laser power. The Raman spectra were acquired in triplicate for each biomass sample. The OPUS software (Bruker Optik GmbH, Ettlingen, Germany) was used for data acquisition and instrument control. A total of 215 spectra were recorded.

### Fourier transform infrared attenuated total reflectance (FTIR-ATR) spectroscopy

The HTS-FTIR analysis showed an increase in saccharide content in *M. alpina* (MA) and *M. hyalina* (MH) when growing at high C/N. This observation motivated further analysis, including FTIR-ATR spectra acquisition since the absorbance at lower wavenumbers, where saccharide peaks are typically located, is more pronounced than in HTS-FTIR (transmission). This enhanced sensitivity is attributed to the penetration depth of the evanescent wave in ATR-FTIR, which is inversely proportional to the wavenumber. Therefore, at lower wavenumbers, the penetration depth is greater, increasing the interaction between the IR light and the sample, and consequently resulting in higher absorbance. The ATR-FTIR spectra pure reference saccharides (chitin, β-glucan, glycogen and trehalose) were also acquired for comparison and discussion.

The spectra were acquired using an aforementioned Vertex 70 FTIR spectrometer (Bruker Optik GmbH, Germany) with a single reflectance attenuated total-reflectance High-Temperature Golden gate ATR Mk II accessory (Specac, United Kingdom) equipped with a horizontal ATR diamond prism with a 45˚ angle of incidence. The *M. alpina* and *M. hyalina* freeze-dried biomass samples were homogenized with the help of an agate pestle prior FTIR-ATR spectra acquisition. Each processed biomass sample was placed on the surface of the ATR diamond and pressed against it with the help of the pressure gate accessory. The spectra acquisition consisted of 32 scans with a spectral resolution of 4 cm^− 1^, and digital spacing of 1.928 cm^− 1^, over the range of 4000 to 600 cm^− 1^, using Blackman-Harris 3-Term apodization. Three technical replicates were acquired for each sample. The OPUS 8.2 software (Bruker Optik GmbH, Germany) was used for data acquisition and instrument control.

### Spectral preprocessing and data analysis

The FTIR-HTS spectra of fungal biomass were preprocessed by data truncation from 3650 to 700 cm^− 1^ and extended multiplicative signal correction (EMSC) (an MSC model extended by linear and quadratic components and polynomial order) [35, 36]. The spectra were analyzed using principal component analysis (PCA) to facilitate visualization and interpretation of the effects of varying C/N ratios and H_2_O_2_ concentrations across all tested strains. The spectra preprocessing and PCA were carried out using Orange Data Mining version 3.33.0 (University of Ljubljana, Slovenia) [37, 38].

Additionally, for the analysis of oxidative damage in *L. corymbifera* (LC) samples, the FTIR-HTS spectra were first converted into second derivatives by using SG algorithm (polynomial 2, window size 15, derivative order 2), followed by truncation from 3650 to 700 cm^− 1^, correction using EMSC, and finally vector normalization.

For comparison of ATR-FTIR spectra from *M. hyalina* (MH) and *M. alpina* (MA) with different reference chemicals, the spectra were first smoothed by using SG algorithm (polynomial 2, window size 11, derivative order 0), followed by truncation from 1800 to 800 cm^− 1^, and finally band area normalization (1546 –1538 cm^− 1^, amide II band C-N stretching and N-H bending vibration in amides, which is predominantly associated with proteins).

Raman spectra of *M. circinelloides* strains (MC and MCVI) for PCA were first converted into second derivatives by using SG algorithm (polynomial 2, window size 17, derivative order 2), followed by truncation from 3200 to 900 cm^− 1^, and finally, vector normalization. Additionally, for comparison with a high carotene content biomass, the spectra were first smoothed by using SG algorithm (polynomial 2, window size 17, derivative order 0), followed by truncation from 1800 to 900 cm^− 1^, rubber band correction and EMSC.

MIC and biomass concentration at sub-lethal H_2_O_2_ doses were analyzed and visualized with Microsoft Excel.

### Microscopy image acquisition

Microscopy was conducted on the mycelium of *M. hyalina* (MH) and *M. alpina* (MA). This analysis was motivated for the same rationale as the ATR-FTIR measurements previously described. Two staining techniques were used to validate and confirm the nature of the increased saccharide-related spectral intensities observed in these species. Lugol’s staining solution was used to visualize glycogen accumulation [39]. Lugol’s stain reagent was prepared with a concentration of 10% I^+^ (w/v) by mixing 1 g of KI and 0.5 g of I_2_ in 10 mL of deionized water. A drop of Lugol’s reagent was added to a drop of hyphae suspension over a microscope slide and covered with a glass cover. The samples were visualized using a Leica DM6 B (Leica, Germany) widefield microscope. Calcofluor white stain solution (calcofluor white M2R, 1 g/L, Evans blue, 0.5 g/L) (Merck, Germany, cat. no. 18909) was used to stain β-glucans and chitin to visualize the thickness of the cell wall. A drop of hyphae suspended in water was placed in a special glass slide for inverted fluorescence confocal microscope. This was followed by the addition of a drop of 10% KOH and a drop of 10-fold diluted calcofluor white solution, which were then mixed. The samples were visualized using a Leica TCS SP5 (Leica, Germany) confocal microscope system equipped with a 405 nm laser with acquisition set with a bandwidth 440–506 nm.

## Results and discussion

### Tolerance to hydrogen peroxide– MIC and MFC

The aim of this experiment was to determine the strain’s tolerance to H_2_O_2_ and their compatibility with typical concentrations used to enhance lignocellulose saccharification with LPMO-containing cellulase cocktails. Additionally, the H_2_O_2_ tolerance levels were analyzed from physiological and ecological perspectives.

**Fig. 1:**
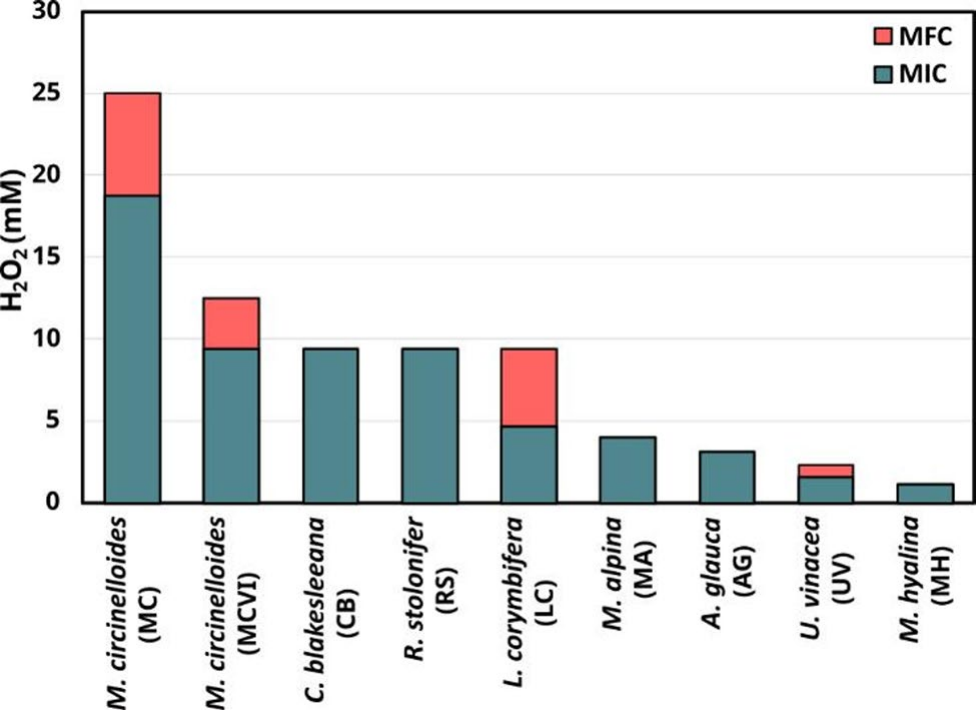
Minimal inhibitory concentration (MIC) values of H_2_O_2_ of the nine Mucoromycota strains evaluated. Both biological replicates showed the same value; hence, error bars are not displayed

The minimal inhibitory concentration (MIC) of H_2_O_2_ on the different fungal strains are shown in Fig. 1. In general, the MIC data aligned with the values of minimal fungicidal concentration (MFC), except for *M. circinelloides* (MC and MCVI), *L. corymbifera*, and *U. vinacea* that showed slightly higher MFC values (Fig. 1 and Additional file 1: Table S2). The MIC values for the entire set of strains ranged approximately from 1 to 19 mM, which is significantly higher than those typically observed in lignocellulose enzymatic saccharification with a continuous H_2_O_2_ feed (0–10 µM) [40] or a single initial dose (240 µM) [12]. Some examples of the defensive mechanism of filamentous fungi towards the oxidative damage of H_2_O_2_ include catalases or the formation of the spore cell wall regulated by the spore coat protein H gene (*cotH*) [41, 42].

According to the MIC results (Fig. 1), the most tolerant strains towards H_2_O_2_ were *M. circinelloides* (MC), *M. circinelloides* (MCVI), *C. blakesleeana*, and *R. stolonifer* with values of 18.75, 9.38, 9.38, and 9.38 mM, respectively. The notable tolerance of *M. circinelloides* is particularly relevant given its potential to cause mucormycosis [43]. This resilience may offer a survival advantage, allowing it to withstand the oxidative stress associated with H_2_O_2_ production by macrophages during the immune response [44]. Indeed, some works have highlighted the over-representation of oxidoreductase activity genes in the transcriptome of *M. circinelloides* compared to other *Mucor* species not associated with mucormycosis [45]. Similarly, *R. stolonifer* is considered a plant pathogen and it has been reported to upregulate protective mechanisms in response to ROS generated by the host plant’s defensive mechanism [46]. Moreover, both *M. circinelloides* and *R. stolonifer* have been reported to accumulate polyphosphates [3] which are known to play a protective role against H_2_O_2_ [47]. The accumulation of polyphosphates could contribute to the increased tolerance of these fungi to oxidative stress. In contrast, *C. blakesleeana* presents a different case. Mucormycosis caused by *Cunninghamella* species is extremely rare and mostly assigned to the species *Cunninghamella bertholletiae* that affects exclusively hematologically compromised patients [48]. Nevertheless, one case of infection with *C. blakesleeana* has been reported in the literature, although it was not confirmed as the cause of the patient’s death [49]. This suggests that the members of the *Cunninghamella* genus may possess shared traits of H_2_O_2_ resistance, potentially enhancing their spore survival under conditions of oxidative stress.

On the other hand, *M. alpina*, *A. glauca*, *U. vinacea*, and *M. hyalina* exhibited relatively low MIC values with 4, 3.13, 1.56, and 1.17 mM, respectively. This observation can be expected since they are species not associated with human or plant pathogenesis. It is noteworthy to mention that the MIC value of *M. alpina* was determined using a mycelium inoculum rather than spores due to the reasons previously discussed. This methodological difference may result in a slightly higher MIC value for *M. alpina*, since mycelium is metabolically more active than spores [50]. Nonetheless, even considering this case, there was a clear distinction between species from genera with pathogenic representatives and those from non-pathogenic groups according to their H_2_O_2_ tolerance This observation aligns with previous findings suggesting that stress tolerance is common among species from genera containing virulent representatives, although not necessarily correlated with virulence itself [51]. Indeed, a similar pattern was observed in this study with *C. blakesleeana*.

### Notes on sublethal H_2_O_2_ exposure under varying C/N ratios

The following sections investigates the combined effects of varying H₂O₂ concentrations and C/N media ratios to determine whether oxidative damage or redox signaling occurs, and if there are combined effects impacting the biochemical composition of fungal biomass.

It is important to consider how H_2_O_2_ diffuses into cells and the concentrations at which it induces oxidative damage or triggers redox signaling. For example, in mammalian cells, it is estimated that the concentrations of H_2_O_2_ follow a gradient of 100 to 650-fold between extracellular and intracellular environments, with higher values in the extracellular environment. In addition, concentrations below 100 nM are typically associated with redox signaling, whereas higher levels promote oxidative distress [52], corresponding to an approximate extracellular H_2_O_2_ concentration of 10 µM to 65 µM. It should be noted that these estimations apply to steady-state conditions, where the concentration remains constant over time. In this study, the sublethal doses of H_2_O_2_ were added at the beginning of cultivation, creating a concentration that likely was not maintained over time due to consumption by the defensive mechanism of each species or redox reactions causing oxidative damage. Additionally, it is known that some metal ions present in the cultivation medium can lead to the consumption of H_2_O_2_ such as iron in the Fenton reaction [53], or H_2_O_2_ production from oxygen catalyzed by copper [54]. However, these reactions are more likely to occur at acidic pH or in the presence of antioxidants, respectively. Additionally, depending on the salt concentrations in the medium, H_2_O _2_ consumption and production may be limited to the nanomolar scale. Moreover, the salt composition and concentration remain identical across all tested conditions. Therefore, if these events do occur, even at an insignificant scale, their effect should be consistent across all conditions. Therefore, although most of the H_2_O_2_ concentrations evaluated in this study exceed the typical ranges where redox signaling operates (Additional file 1: Tab. S1), it does not necessarily imply oxidative damage effects in all cases.

### Effects on biomass production

**Fig. 2:**
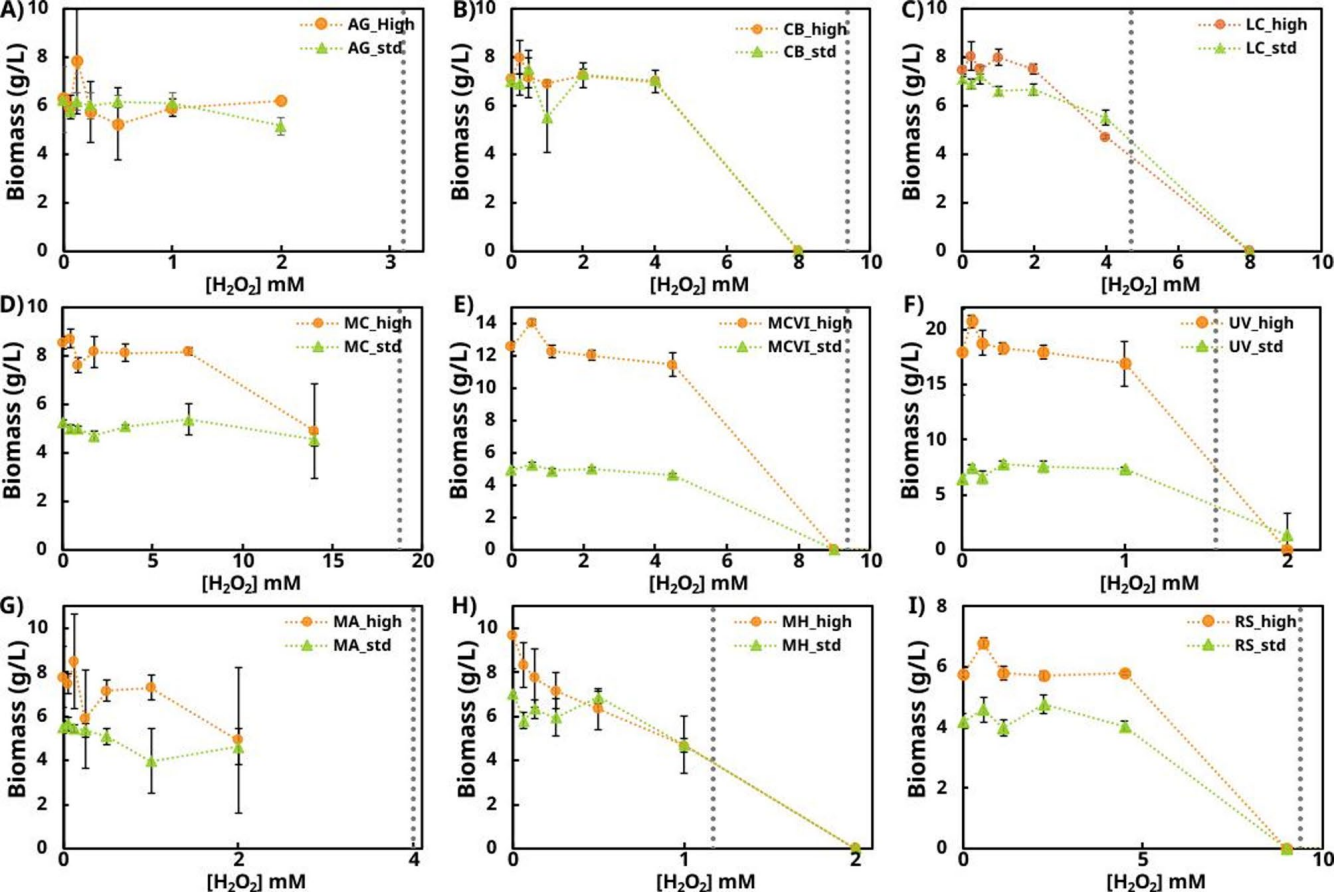
The relation between biomass concentration and H_2_O_2_ concentration for each strain when cultivated in high C/N (89) and standard C/N (18.8) medium. (A) *A. glauca* (AG), (B) *C. blakesleeana* (CB), (C) *L. corymbifera* (LC), (D) *M. circinelloides* (MC), (E) *M. circinelloides* (MCVI), (F) *U. vinacea* (UV), (G) *M. alpina* (MA), (H) *M. hyalina* (MH), and (I) *R. stolonifer* (RS). Grey vertical dash lines mark the corresponding MIC value

As an initial step in evaluating the effects of sublethal H_2_O_2_ exposure, microbial biomass production was determined at different concentrations of H_2_O_2_ (Fig. 2). In general, H_2_O_2_ concentrations around 2-fold lower than the corresponding MIC did not show effects on biomass reduction. Meanwhile, in some cases where higher concentrations were tested a reduction in biomass was detected, such as *L. corymbifera* grown in both C/N media at 4 mM H_2_O_2_ and *M. circinelloides* (MC) in the medium with high C/N at 14 mM H_2_O_2_. Note also that biomass growth was inhibited at slightly lower concentration, 8 mM H_2_O_2_, than the determined in the MIC assay. This can be explained by the discrete nature of MIC values in the microdilution assay. The final concentration range tested was between 9.375 and 6.25 mM, and since inhibition was observed at 8 mM, the result remains consistent with the MIC assay. These results suggest that, even at the H_2_O_2_ concentrations typically used to enhance saccharification (1–240 µM), adverse effects on biomass production are unlikely to occur.

On the other hand, biomass values differed according to the C/N conditions, with the highest values detected under high C/N. This increase in biomass reflected a significant metabolite accumulation driven by the carbon-nitrogen imbalance of the high C/N ratio medium. The biggest differences between media were found for *U. vinacea*, and the two *M. circinelloides* strains (MCVI and MC). Additionally, minor differences in biomass production between C/N conditions were also detected for *M. alpina*, *M. hyalina*, and *R. stolonifer*.

### Effects on the chemistry of the fungal biomass composition investigated by vibrational spectroscopy

**Fig. 3:**
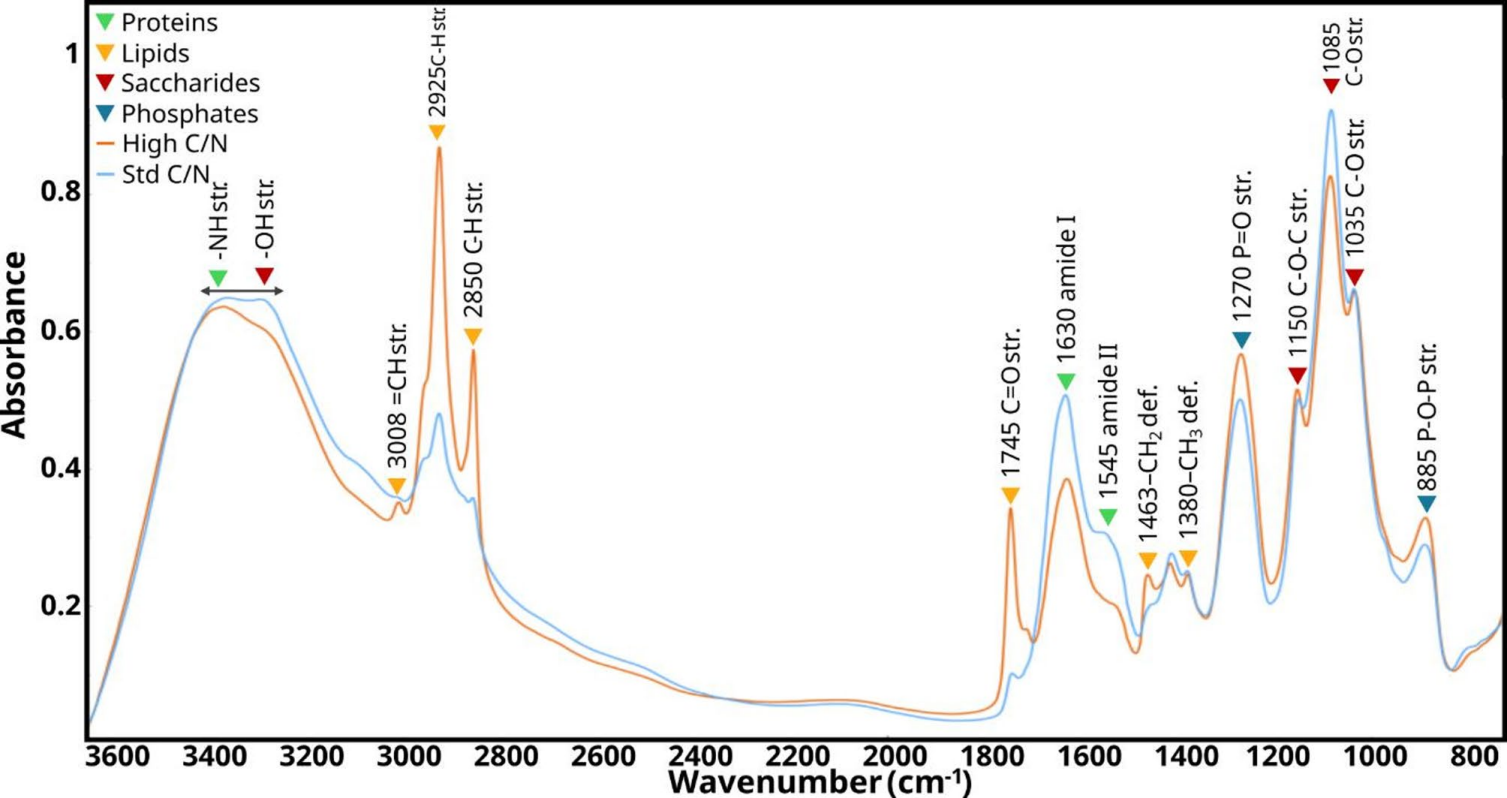
Spectra of *Mucor circinelloides* (MCVI) grown at standard (blue line) and high (orange line) C/N medium without H_2_O_2_. The main peaks and vibrational modes associated with the different metabolites that are typically found in this type of sample (def.: deformation; str.: stretching). The color code designates the class of chemicals with the dominant contribution to the absorbance on the marked bands

FTIR and FT-Raman have been demonstrated to be excellent tools for studying and monitoring biotechnological processes such as fermentation to track changes in media composition and biomass metabolites [25, 33, 55]. These techniques are fast and cost-effective, providing simultaneous information of multiple functional groups, thereby, multiple chemical compounds. These techniques can be used as semiquantitative or quantitative analysis when a proper, but often complex, calibration is done [8, 25, 29]. In this study, vibrational spectroscopy was used as a semi-quantitative technique, which was sufficient to reflect changes in the relative content of major biomolecules. These techniques were completely compatible with the limited biomass amounts of the Duetz cultivation system employing 96-well plates, whereas other and multiple analytical methods could not. Due to the predominance of FTIR in this study, a typical FTIR spectrum from a sample of *M. circinelloides* is shown in Fig. 3, displaying the main peaks of associated metabolites. Note that the dominance of a particular metabolite in a peak does not preclude minor contributions from others.

PCA analysis of the FTIR spectra was conducted to identify and visualize the most significant chemical changes in fungal biomass grown under different C/N and sublethal H_2_O_2_ concentrations. PCA allows the detection of the main sources of variation between the analyzed samples using principal components (PCs) [56]. The PC loading plots are useful to identify the wavenumbers that contribute the most to variability between spectra within a dataset, enabling inference of the biomolecules that cause the differences.

Given the number of different strains and growth conditions, interpreting PCA results in a single plot was complicated since the pronounced variability in some strains and conditions hindered more modest changes in others. The PCA score plot with all the data can be found in Additional file 1: Fig. S1. Therefore, PCA results were plotted separately in three subgroups of strains and using the most descriptive PCs: (1) *M. circinelloides* (MC and MCVI strains), *U. vinacea*, and *R. stolonifer* with PC1 and PC3; (2) *A. glauca*, *L. corymbifera*, and *C. blakesleeana* with PC2 and PC4; and (3) *M. alpina* and *M. hyalina* with PC1 and PC4. Hereafter, the analysis, interpretation and discussion of these subgroups is presented in subsections accordingly.

#### M. circinelloides, U. vinacea and R. stolonifer

**Fig. 4:**
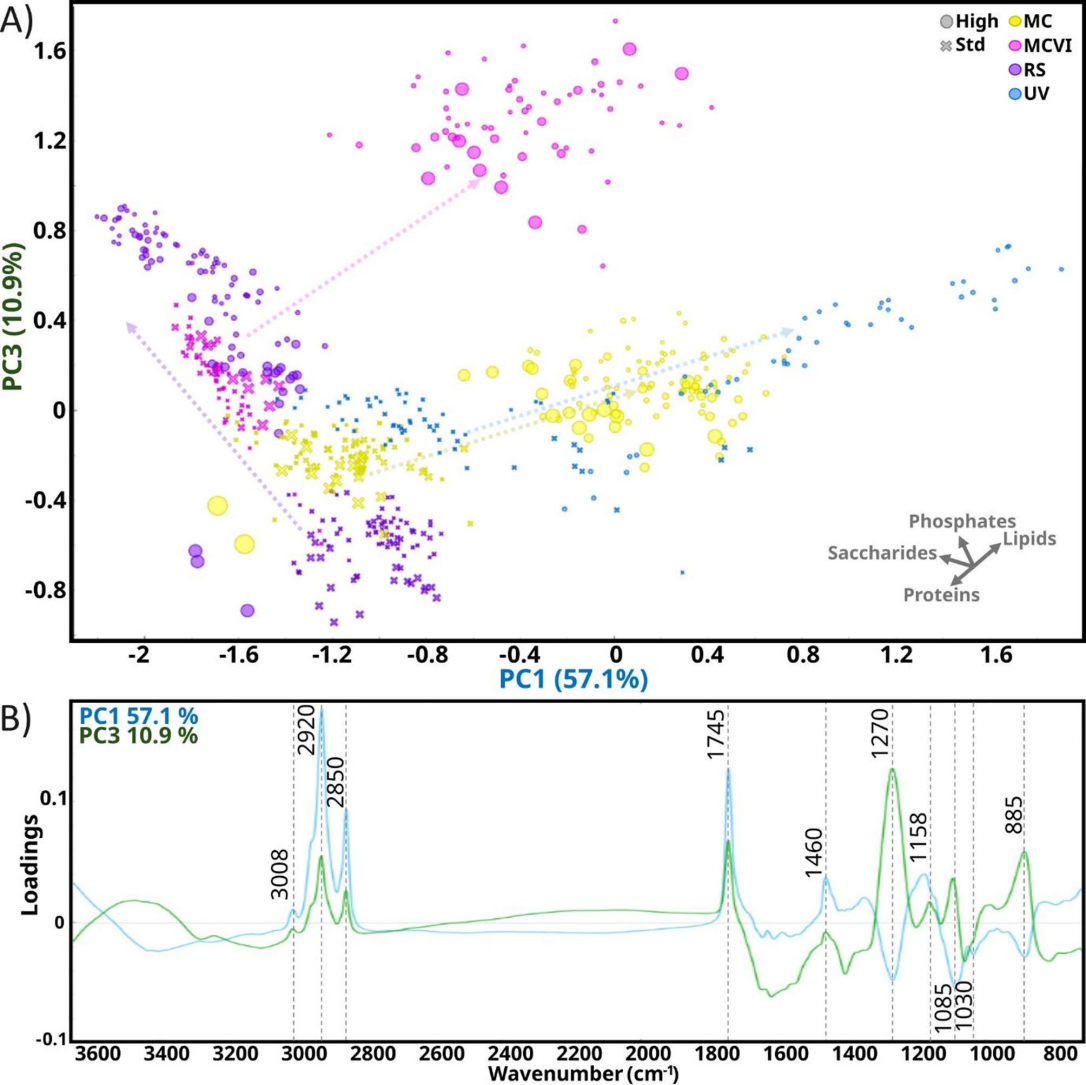
PCA score and loadings plots of FT-IR spectra of *M. circinelloides* (MC and MCVI strains), *R. stolonifer* (RS), and *U. vinacea* (UV) grown at standard (⨯) and high (●) C/N and different concentrations of H_2_O_2_ represented by relative proportional symbol size. (A) score plot using PC1 and PC3 with an explained variance of 57.1% and 10.9%, respectively. Vector axes are provided as an approximate indication of the direction of the relative increase of each metabolite. Color semitransparent dashed-line arrows indicate the progression of each species from standard (18.8) to high (89) C/N conditions. (B) Loadings plot of PC1 and PC3 with the most relevant peaks assigned. The explained variance for the first five PC was: 57.1%, 16.5%, 10.9%, 4.7%, and 3%

The Fig. 4 presents the score and loading plots using PC1 and PC3 for the strains *M. circinelloides* (MC and MCVI), *U. vinacea*, and *R. stolonifer*, with designation of species and C/N conditions. The PC1 loading plot (Fig. 4b) shows major positive loadings related to IR bands associated with lipids, such as 3008, 2920, 2850 cm^− 1^ (= C-H stretching, C-H stretching from -CH_3_; C-H stretching from -CH_2_ respectively), 1745 cm^− 1^ (C = O stretching, triglyceride esters), and 1460 cm^− 1^ (-C-H bending); moderate negative loadings from bands associated with phosphates, 1270 cm^− 1^ (P = O stretching) and 885 cm^− 1^ (P-O-P); and saccharides, 1085 cm^− 1^ and 1030 cm^− 1^ (C-O stretching hydroxyl groups of carbohydrates). On the other hand, the PC3 loading plot showed major positive loadings associated with phosphates and lipids, assigned by the respective and previously indicated bands.

Considering the clusters formed according to C/N conditions for *M. circinelloides* (MC and MCVI) and *U. vinacea*, from standard to high C/N conditions, the data were displayed along the positive direction of PC1 and PC3. In contrast, for *R. stolonifer*, the data were displayed primarily along the PC3 positive direction and, to some extent, along the PC1 negative direction. According to the loading plot interpretation provided above, under high C/N conditions, *M. circinelloides* (MC and MCVI) and *U. vinacea* accumulated primarily lipids, and moderately phosphates, whereas *R. stolonifer* accumulated mainly phosphates and slightly lipids. According to the literature on these species grown at similar high C/N, the lipid signals are predominantly attributed to the accumulation of triacylglycerols (TGAs) in lipid bodies [57, 58] while the phosphate signals can be attributed in their majority to polyphosphate accumulation [3]. It is worth noting that these studies validated spectral analysis and interpretation using complementary analytical techniques, such as solid-state NMR, UV-VIS spectrometry, transesterification and GC-FID, among others.

On the other hand, no distinct clustering on the PCA score plot according to H_2_O_2_ conditions was observed. However, a slight trend was noted for both *M. circinelloides* strains (MC and MCVI) under high C/N, where biomass samples grown at the highest H_2_O_2_ concentrations were positioned towards lower PC1 and PC3 values, reflecting a reduction in lipid and polyphosphate accumulation. This observation suggests a potential adverse effect due to H_2_O_2_ that agrees with the biomass decrease found at the highest sublethal H_2_O_2_ concentration (Fig. 2). Similarly, in *R. stolonifer*, a slight effect was observed under high C/N conditions, where samples grown at the highest H_2_O_2_ concentrations appeared at lower PC3 values, indicating reduced polyphosphate accumulation. Thus, noticeable changes in biomass chemical composition were observed only near the corresponding MIC value of H_2_O_2_.

**Fig. 5:**
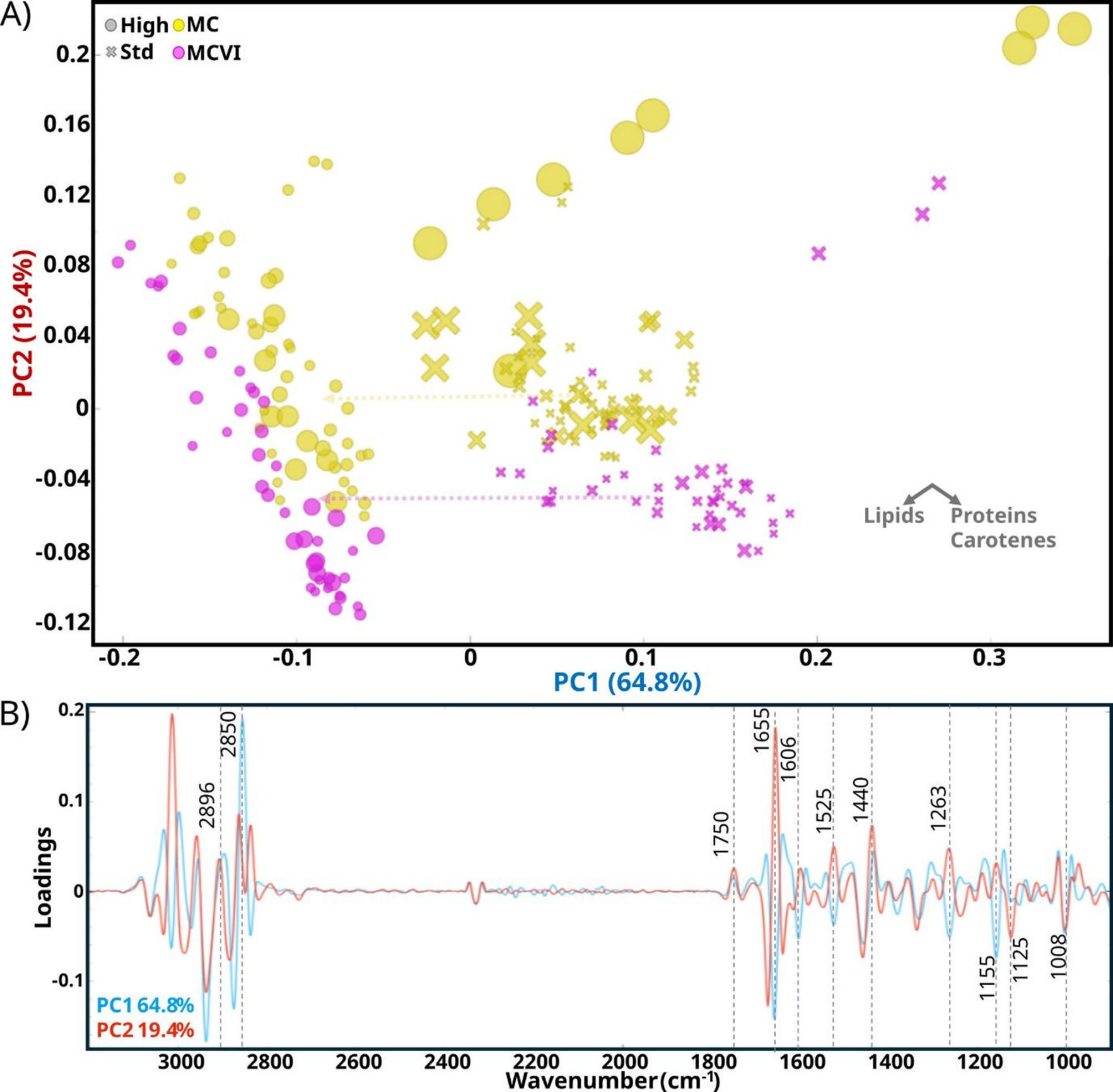
PCA score and loadings plots of Raman spectra of *M. circinelloides* (MC and MCVI strains), grown at standard (⨯) and high (●) C/N and different concentrations of H_2_O_2_ represented by relative proportional symbol size, with the bigger size, the highest concentration. (A) score plot using PC1 and PC2 with an explained variance of 64.8% and 19.4%, respectively. The spectra were converted into second derivatives, and thus high negative intensity values correspond to the high Raman intensity. Vector axes are provided as an approximate indication of the direction of the relative increase of each metabolite. Color semitransparent dashed-line arrows indicate the progression of each species from standard (18.8) to high (89) C/N conditions. (B) Loadings plot of PC1 and PC2 with the most relevant peaks assigned. The explained variance for the first 5 PC was: 64.8%, 19.4%, 5.5%, 2.9%, and 1.6%. For interpretation, note that spectra were preprocessed with second derivative and the direction of the peak intensities is displayed upside down

Additionally, *M. circinelloides* strains (MC and MCVI) are known to produce carotenes [29], that have a role as ROS neutralizers [59]. Therefore, it could be expected an increase in carotenes with varying H₂O₂ concentrations, as it has been observed in some studies with *Neurospora crassa* [60]. Carotenes are pigments consisting of isoprene units, and their conjugated double bonds promote resonance Raman effects that result in a high-sensitivity detection by the peaks at 1525, 1155, and 1005 cm^− 1^ (C = C stretching from polyene chain, C-C stretching and -CH deformation, and C-CH_3_ deformation, respectively) [29, 61]. From the PCA analysis of FT-Raman spectra (Fig. 5a), clustering of samples was observed only by C/N condition, while no clear patterns correlated with the sublethal concentrations of H_2_O_2_ were found. According to the PC1 and PC2 loadings plots (Fig. 5b), the main wavenumbers contributing to the clustering by C/N condition were associated with the relative content of lipids and proteins. PC1 was highly positively correlated with wavenumbers related to proteins, such as 1655 (-C = O stretching amide I), 1606 (C = C stretching phenyl ring of aromatic amino acids), and 1261 cm^− 1^ (C-NH deformation, amide III), and slightly positively correlated with the most intense carotene related peaks 1525 and 1125 cm^− 1^, while the opposite applied to PC2. On the other hand, PC1 was highly negatively correlated with lipid-associated wavenumbers such as 2896 (-CH_3_ stretching), 2850 (-CH_2_ stretching), 1750 (C = O stretching), 1440 (CH_2_ and CH_3_ deformation), and the opposite for PC2. Note that Raman has more sensitivity towards apolar groups while FTIR for polar groups. Therefore, highly polar bonds, such as in orthophosphates produce very weak Raman signals, so their variability between samples was not captured as effectively as in FTIR [29].

To sum up, samples grown at high C/N were clearly chemically different compared to the samples grown at standard C/N. Only samples from *M. circinelloides* (MC) grown at high C/N at the highest H_2_O_2_ sublethal concentration (14 mM) had composition similar to the standard C/N samples, due to the inhibiting and deleterious effects previously commented. Overall, the PCA indicated that the main difference in biomass was the relative content of lipids, higher in high C/N, and proteins, higher in standard C/N. Also, higher relative content of carotenoids was observed for the samples grown in standard C/N. It is noteworthy to mention that visually color differences were not detected at the naked eye between samples, and the differences detected by FT-Raman spectroscopy were minimal. For comparison, a stack graph of Raman spectra including both the samples of this study and high β-carotene content samples validated by HPLC-UV analysis from the study of Dzurendova et al. [29] is provided in Additional file 1: Fig. S2. These results suggest that in high C/N conditions, metabolites like lipids, polyphosphates, and saccharides were accumulated while β-carotene content remained unchanged.

Finally, it must be noted that in the study of Iigusa et al. [60] the enhancement of β-carotene production by H_2_O_2_ was assayed in combination with other factors such as air and light exposure. H_2_O_2_ enhanced β-carotene production slightly only when light was present. In our study, fermentation experiments were conducted using 96-well microplates of relatively thick polypropylene plastic, lids that are totally opaque to light, a shaker incubator without a light source, and a H_2_O_2_ dosage present from the start of inoculation. Therefore, similarly as reported by Iigusa et al., H_2_O_2_ alone in absence of light did not trigger carotene production, in *M. circinelloides* in our case.

#### A. glauca, C. blakesleeana, and L. corymbifera

**Fig. 6:**
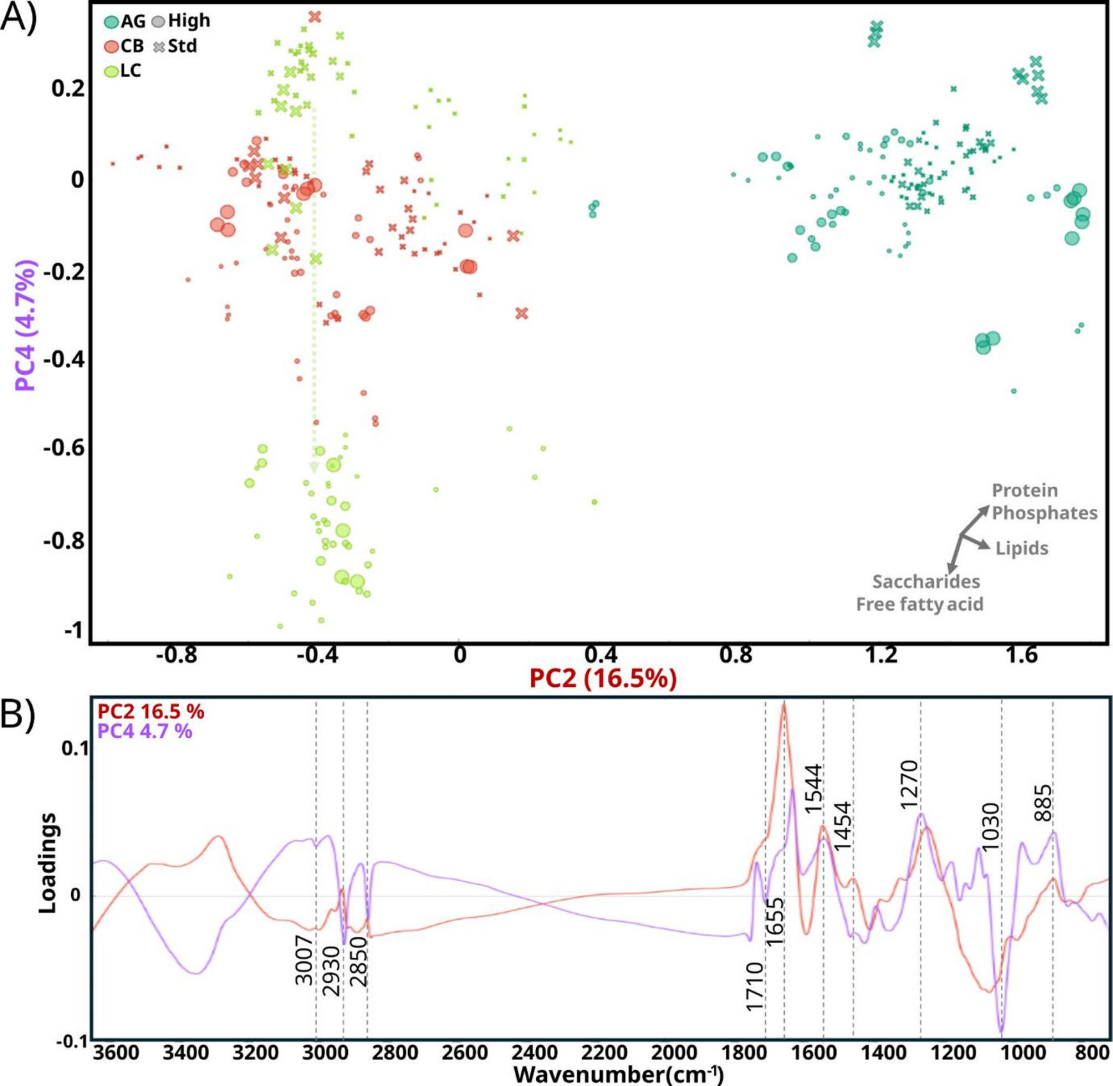
PCA analysis of FT-IR spectra from *A. glauca* (AG), *C. blakesleeana* (CB), and *L. corymbifera* (LC) grown at standard (⨯) and high (●) C/N and different concentrations of H_2_O_2_ represented proportional symbol size. (A) score plot of PC2 and PC4, with an explained variance of 16.5 and 4.7% respectively. A vector axis is provided as an approximate indication of the directions of the relative increase of each metabolite, and a color transparent dash-line arrow is added to indicate the progression from standard to high C/N. (B) Loading plot of PC2 and PC4 with corresponding chemical-related peaks marked. The explained variance for the first 5 PC was: 57.1%, 16.5%, 10.9%, 4.7%, and 3%

PCA results for the strains *A. glauca*, *L. corymbifera*, and *C. blakesleeana* were visualized separately using PC2 and PC4 (Fig. 6a). The PC2 loading plot (Fig. 6b) showed major positive loadings from protein-associated signals at 1655 cm^− 1^ and 1544 cm^− 1^, attributed to C = O stretching and C-NH deformation amide I and II, respectively; moderate negative loadings associated with phosphate bands at 1270 cm^− 1^ and 885 cm^− 1^, and minor positive loadings from lipid-associated signals at 3007, 2930, 2850, 1454 cm^− 1^ from aliphatic -CH. Considering that the signal at 1745 cm^− 1^ associated with TGAs’ esters was not clear in the PC2 loadings, it can be interpreted that PC2 reflects small differences in length and unsaturation of lipids rather than relative acylglycerols content. On the other hand, the PC4 loading plot (Fig. 6b) showed major negative loadings associated with saccharide-related bands such as at 1150 cm^− 1^ and 1030 cm^− 1^, moderate positive loadings associated with phosphate-related bands at 1270 cm^− 1^ and 885 cm^− 1^, protein-related bands at around 1655 cm^− 1^ and 1544 cm^− 1^ and minor negative loadings associated with lipid-related peaks at 3007, 2930, 2850 and 1710 cm^− 1^ (C = O stretching from free fatty acids). The PCA score plot for these three species revealed a clear separation according to PC2. Specifically, spectra of *A. glauca* were separated from *C. blackesleeana* and *L. corymbifera*, being positioned towards PC2 positive values. According to the PC2 loading plot described above, it can be interpreted that *A. glauca* had a higher content of proteins and phosphates, a lower amount of saccharides, and a slightly higher amount of longer and unsaturated fatty acids compared to *C. blakesleeana* and *L. corymbifera*. On the other hand, clustering according to C/N conditions was only observed for *L. corymbifera*. From standard to high C/N conditions, the samples of *L. corymbifera* moved towards negative PC4 values, indicating higher content of saccharides and free fatty acids under higher C/N conditions. When it comes to H_2_O_2_ effects, the PCA plot showed only slight trends for *A. glauca* grown at the highest H_2_O_2_ concentrations. The samples of *A. glauca* from these H_2_O_2_ conditions were positioned further to the positive PC2 scores, indicating an increase in protein-related signals. Given that the nitrogen source in the medium was limited, these results were likely to be due to a relative decrease in other biomolecules, such as lipids, polyphosphates, and saccharides. This pattern aligns with the inhibitory effects of H_2_O_2_, where oxidative stress might suppress the synthesis or accumulation of these biomolecules.

#### M. alpina and M. hyalina

**Fig. 7:**
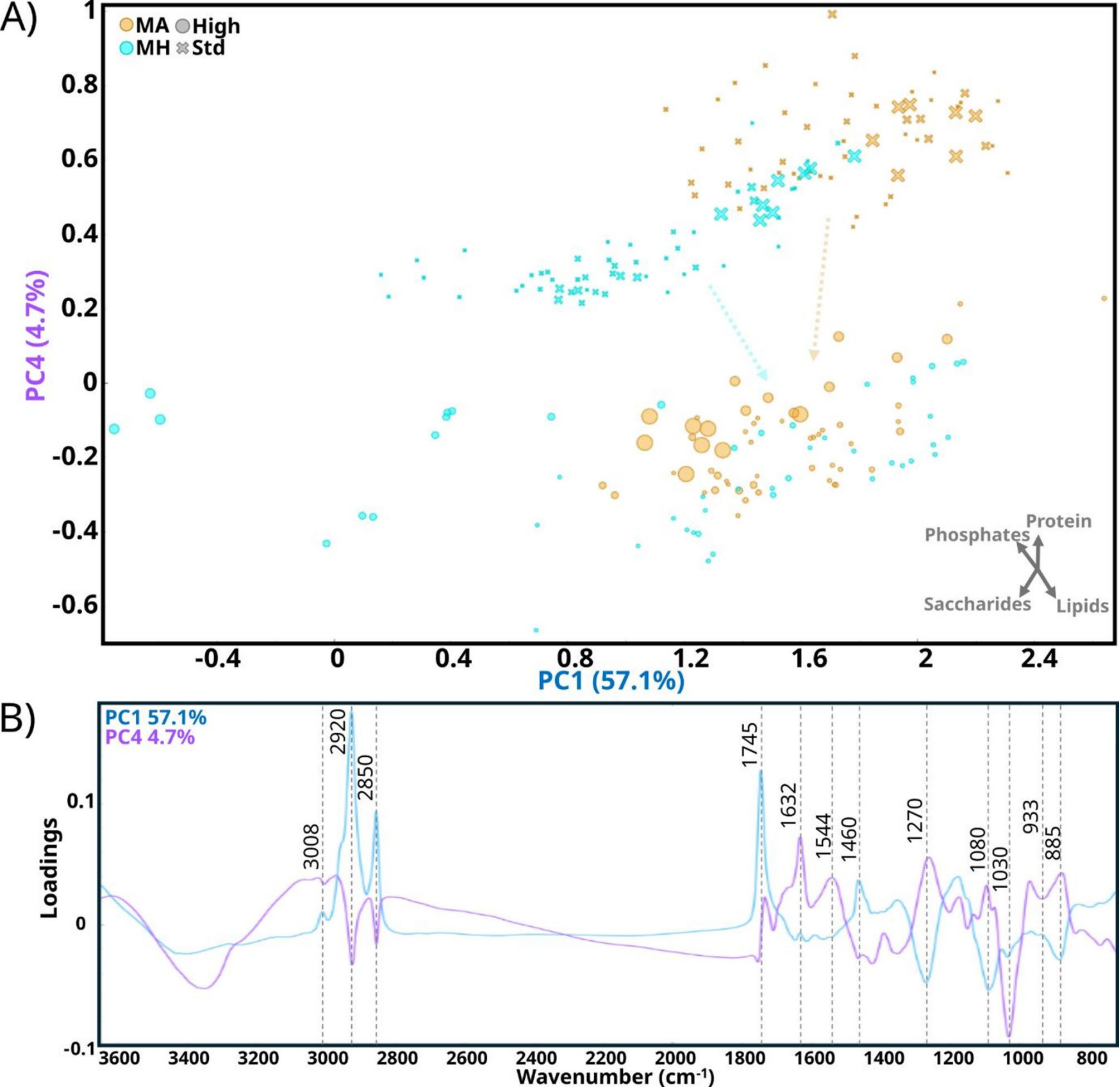
PCA analysis of FT-IR spectra from *M. alpina* and *M. hyalina* grown at standard (⨯) and high (●) C/N and different concentrations of H_2_O_2_ represented by symbol size. (A) score plot of PC1 and PC4, 57.1 and 4.7% of the explained variance, respectively. A vector axis is provided as an approximate indication of the direction of the relative increase of each metabolite. (B) Loading plot of PC1 and PC4 with corresponding chemical-related peaks marked. The explained variance for the first 5 PC was: 57.1%, 16.5%, 10.9%, 4.7%, and 3%

Lastly, the PCA score plot of *M. hyalina* and *M. alpina*, using PC1 and PC4, revealed a distinct separation of the samples grown under standard and high C/N conditions (Fig. 7a). Analogous to the previous discussions, the PC1 loading plot showed major positive loadings associated with lipids and moderate negative loadings associated with phosphates and saccharides. On the other hand, the PC4 loading plot shows major negative loadings associated with saccharides, minor negative loadings associated with lipids, and moderate positive loadings associated with phosphates and with proteins (Fig. 7b).

Under standard C/N conditions, the two *Mortierella* species were primarily separated along PC1, due to differences in lipid content, the shape of amide I and II bands, and differences in saccharides composition driven by the differences in proportion of -COH associated peaks at 1080 and 1032 cm^− 1^. In contrast, when both species were grown under high C/N condition, the distinction between these species was less pronounced, with the samples appearing closer together in the PCA score plot. The distinction between samples grown under high and standard C/N conditions remained evident and was primarily captured by PC4. From standard to high C/N conditions, the samples of both species shifted toward more negative values of PC4, indicating a decrease in proteins and phosphates while an increase in lipids and saccharides. However, due to the nitrogen limitation in the medium, it is reasonable to consider the amide II band as a stable internal reference band for comparing spectra of the same fungal species grown at different conditions. When normalized to amide II band, samples grown under high C/N conditions, effectively, showed higher levels of lipids and saccharides (Additional file 1: Fig. S3). Interestingly, the phosphate-related bands in these *Mortierella* species were neither as sharp nor intense as those observed in other species known for extensive polyphosphate accumulation, such as *M. circinelloides* or *R. stolonifer* [3] (Additional file 1: Fig. S4). In the normalized spectra of both *Mortierella* species, no clear increase in phosphate peaks was detected under high C/N conditions. This suggests that, in these species, the phosphate-related peaks were primarily associated with the content of nucleotide-based molecules rather than polyphosphates. Given that the proportion of DNA and RNA in the overall biomass remains relatively stable, their associated phosphate bands may have relatively low intensity due to the increase of bands associated with the accumulation of lipids and saccharides under high C/N conditions. Further research into the quantity and molecular forms of the phosphate accumulated in *Mortierella* species would be valuable.

Interestingly, the increase in saccharides at high C/N was marked mainly by the peak at 1030 cm^− 1^ with less influence from the peak at 1080 cm^− 1^. The 1080 cm^− 1^ is also weakly influenced by phosphates (P = O asymmetric stretching) that did not increase with higher C/N, explaining this observation. It is known that glycogen and trehalose are saccharides commonly used for energy storage in fungi [62]. Indeed, it has been reported that *M. alpina* can accumulate up to 20–30% of total saccharides (in cell wall and cytosol) and 5–7% intracellular saccharides (mainly glucose and trehalose) during lipid accumulation fermentation conditions [63, 64]. Thus, accumulation not only of trehalose and glucose, but also of glycerol, was reported to play a role in adaptation to cold temperatures in *Mucor psychrophilus* and *Mucor flavus* [65, 66]. Particularly, *Mortierella* species are often cold-adapted and isolated in alpine, Antarctic, and Arctic environments [67–70], where other representatives of Mucoromycota are absent. Therefore, the saccharide/polyol metabolite accumulation could be related to their lifestyle.

**Fig. 8:**
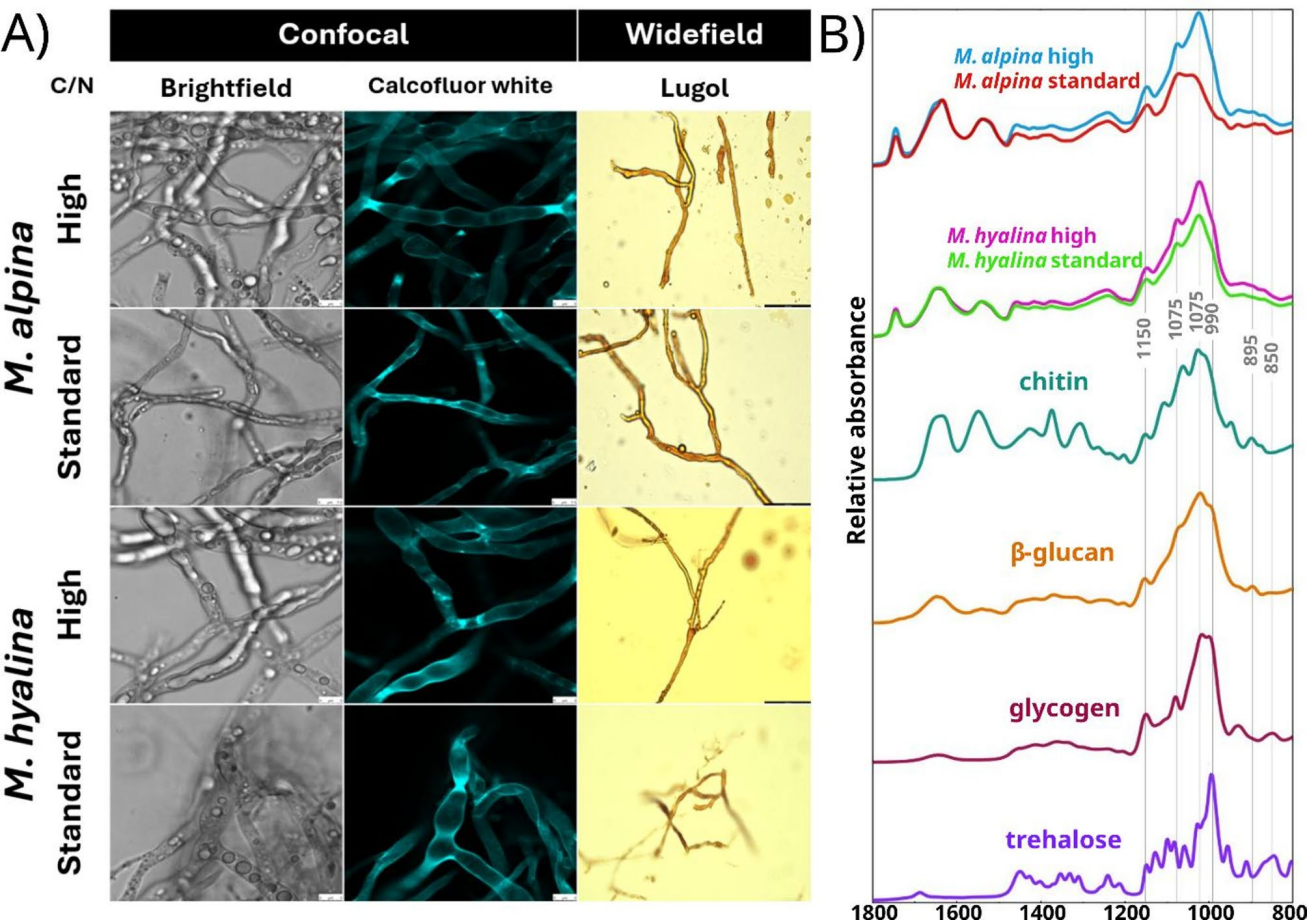
(A) Microscopy images of *M. alpina* (MA) and *M. hyalina* (MH) in brightfield, with calcofluor white stain and Lugol’s stain for standard (18.8) and high (89) C/N medium. (B) Stacked ATR-FTIR spectra of *M. alpina* and *M. hyalina* grown in high and standard C/N medium without H_2_O_2_ compared with different saccharides such as α-chitin, β-glucan, glycogen, and trehalose. The spectra were preprocessed by data truncation from 1800 –800 cm^-1^, Savitzky-Golay filter (window 11, polynomial order 2). In addition, *M. alpina* and *M. hyalina* samples were normalized by the amide II band

A comparative analysis involving pure reference saccharides was performed to provide the most likely type of metabolite accumulated. The spectra of both *M. alpina* and *M. hyalina* grown at high and standard C/N were compared with the reference spectra of different saccharides (Fig. 8). These reference chemicals represented not only carbon and energy storage saccharides, such as trehalose and glycogen, but also structural saccharides, such as chitin and β-glucan. The bonds between monomers of trehalose and glycogen are α-O-glycosidic type, while chitin and β-glucans are β-O-glycosidic type. The anomeric carbon region from 900 to 800 cm^− 1^ can be used to differentiate β- and α-O-glycosidic bonds by specific peaks at 895 cm^− 1^ and 850 cm^− 1^, respectively [71]. Unfortunately, these peaks are relatively weak, and phosphates also interfere in this region, making the discrimination of α and β polymers impossible.

Other stronger peaks were also considered. For instance, the main C-O stretching peak in trehalose is around 990 cm^− 1^, while for glycogen, chitin, and β-glucan, it is around 1020 cm^− 1^. However, relative changes between 990 cm^− 1^ and 1020 cm^− 1^ were not observed under high C/N conditions for none of the *Mortierella* species, which suggest a non-particular specific enrichment of one over the other. On the other hand, if the intensity increases in the region 900 to 1100 cm^− 1^ were due to chitin, it would be accompanied by a proportional increase in the amide I and II band, too. In such cases, normalization of spectra by the amide I or II band should remove the observed increases in carbohydrate-associated bands in the range 1100 –900 cm^− 1^. However, after normalization by amide II, the increase in the carbohydrate-associated bands under high C/N conditions was still observed (Fig. 8). Additionally, comparing the samples in the confocal fluorescence microscope stained with calcofluor white, we did not observe notable increases in cell wall thickness between samples grown at standard and high C/N medium (Fig. 8). A microscopy picture of *C. blakesleeana*, a species from a genus well-known for cell wall production [72, 73] is provided in Additional file 1: Fig. S5, as proof of the effectiveness of calcofluor white staining for visualization of cell wall thickness. Therefore, an increase in chitin or cell wall glucans was excluded.

From Lugol’s staining, no evident differences in dark brown coloration, indicative of glycogen accumulation, were observed. This staining method for glycogen was previously used in some publications but with samples with big differences in glycogen content, such as between hyphae and survival structures (spores, chlamydospores, etc.) [74–76]. Therefore, Lugol’s staining results suggested that glycogen levels were not significantly higher than under standard C/N conditions, and other molecules likely contributed more to the observed spectral intensities. Separately, we found Lugol’s staining to be quite effective for detecting chlamydospores in *M. hyalina* biomass grown on standard C/N medium (Additional file 1: Fig. S6). It might be that the total depletion of nutrients in the standard C/N medium motivated the formation of chlamydospores, which is a survival structure.

To sum up, our analysis suggests that the accumulated saccharides in *Mortierella* were primarily intracellular, although their precise nature remained elusive. More detailed analysis could be achieved using methods, such as band deconvolution in specific spectral regions [77], or more complex wet-chemical analysis, as reported by Danilova et al. [66]. However, such investigations were beyond the scope of this study.

#### General observations

Generally, no enhancement in lipid accumulation was detected in any of the fungal strains across their corresponding range of sublethal H_2_O_2_ concentrations. This contrast with the case of several microalgae species, grown autotrophically, where ROS signaling induced by H_2_O_2_ stress is widely reported to enhance the accumulation of neutral lipids [78–80]. It is also noteworthy to mention that it could not be discarded also some possible effects in other setups, for example a pulsed addition of H_2_O_2_ during late logarithmic or stationary growth phase, which could trigger the production of certain metabolites. However, this was out of scope of this study since we focused on the presence of H_2_O_2_ from the beginning of the fermentation, as expected in H_2_O_2_ assisted SSF.

### Detection of oxidative damage

Spectra from each strain were analyzed according to the sublethal H_2_O_2_ concentrations to identify oxidative damage not detected by PCA. Oxidative damage effects were only perceived in the *L. corymbifera* strain. The detailed spectral analysis revealed some changes in the amide I and II bands in correlation with the H_2_O_2_ concentration in *L. corymbifera* grown under standard C/N, while not at high C/N. Although glucose can be oxidized by H_2_O_2_, this reaction rate is very slow at 25 °C in the absence of a catalyst [81]. Therefore, it is unlikely that the higher amount of glucose in the high C/N medium could deplete H_2_O_2_ in significant amounts promoting lower H_2_O_2_ exposure. Therefore, the observed differences between standard and high C/N were most likely due to the metabolic processes of the fungus.

It is known that glucose-6-phosphate dehydrogenase is a pivotal enzyme in the generation of NADPH in the pentose phosphate pathway, relevant for lipogenesis. Indeed, this enzyme has been reported to be overexpressed during the lipogenesis of *M. circinelloides* cultivated in high C/N medium [6, 82]. On the other hand, this enzyme and the generation of NADPH is also essential for maintaining the glutathione antioxidant system [83], crucial to maintain the redox balance. Therefore, it is possible that at high C/N the oxidative damage of proteins triggered by H_2_O_2_ was also prevented by the role of glucose-6-phosphate dehydrogenase.

**Fig. 9:**
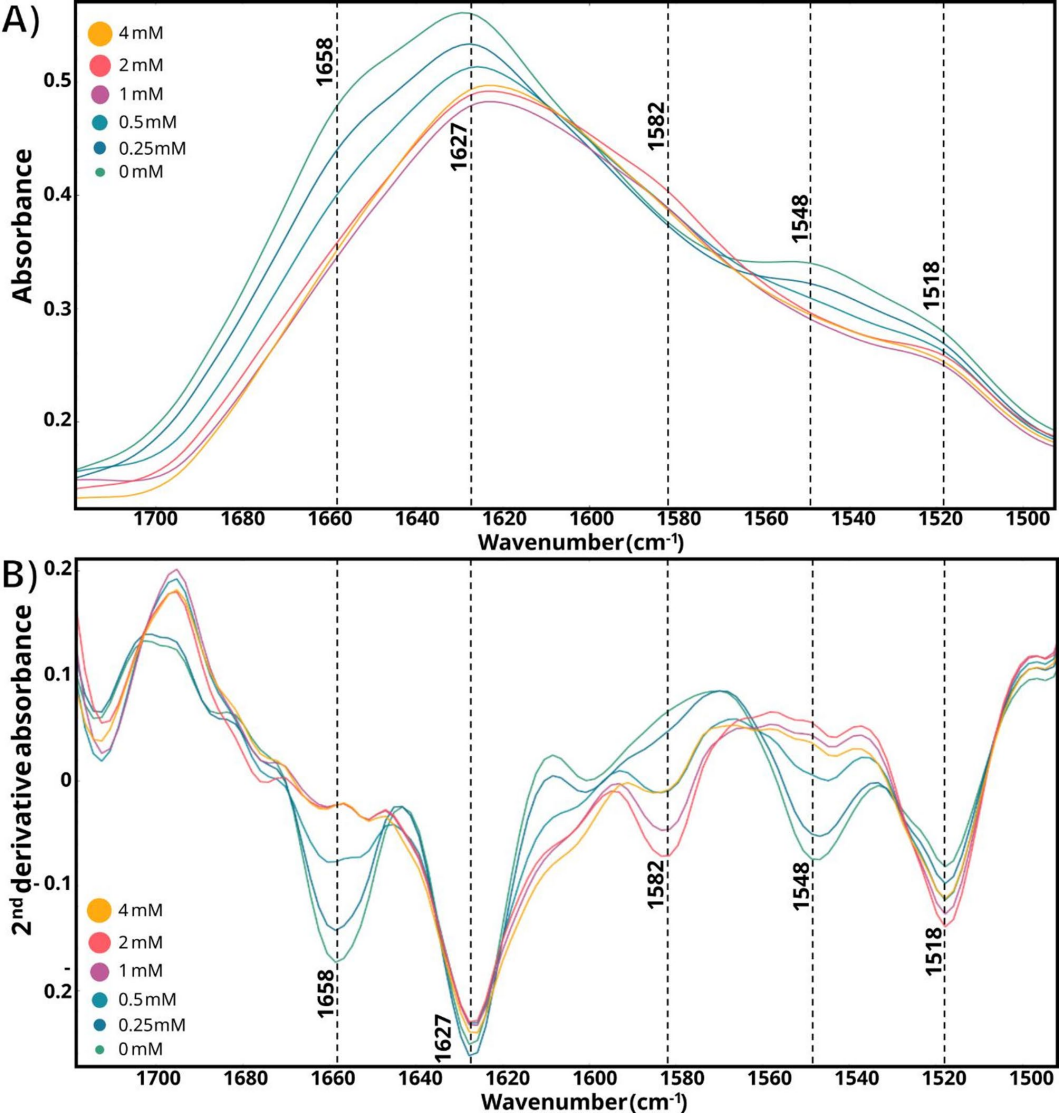
Amide I and II regions of *L. corymbifera* grow at standard C/N (18.8) medium under different H_2_O_2_ concentrations. (A) Absorbance and (B) second derivative absorbance spectra with relevant wavenumbers associated with α and β conformations in proteins

Back to the topic, when *L. corymbifera* was grown at standard C/N conditions, notable alterations were observed in the amide I and II bands positively correlated with increasing H_2_O_2_ concentrations (Fig. 9a). These changes involved both the shape and intensity of the amide bands.

In terms of band shape, a decrease in the shoulder around 1658 cm^− 1^ accompanied by shift in maximum intensity towards 1628 cm^− 1^ was observed. This narrowing may indicate an increase in random and β-sheet secondary protein conformations at the expense of α-helix conformation. This is supported by a relative decrease in peaks at 1658 and 1545 cm^− 1^ and an increase in peaks at 1625 and 1518 cm^− 1^. This was even better visualized by applying a second derivative preprocessing (Fig. 9b). Typically, contributions in amide I and II bands at higher wavenumbers (around 1651 and 1545 cm⁻¹, respectively) are associated with α-helices and random coil conformations [84], while lower wavenumbers (around 1625 and 1520 cm^− 1^, respectively) are associated with intermolecular β-sheet structure interactions [85]. The denaturation and aggregation of proteins into highly ordered β-sheet supramolecular structures promote these changes [86–88]. Indeed, ROS, such as H_2_O_2,_ can facilitate denaturation and aggregation of proteins via mechanisms such as methionine oxidation [89].

Regarding the intensity, both amide I and II bands decreased in intensity, while the peaks at 1585 and 1411 cm^− 1^ increased. The 1585 and 1411 cm^− 1^ peaks can be associated with symmetric and asymmetric stretching of carboxylate free groups (COO^−^), respectively. In addition, the increase in the peak at 1518 cm^− 1^ can be assigned to the NH_3_^+^ (scissoring) group of *N*-terminal in proteins. These observations agree with the breakdown of peptide bonds during protein hydrolysis [90]. Therefore, these results suggested an accumulation of intracellular proteins with oxidative damage, consistent with previous works of high H_2_O_2_ exposition in filamentous fungi [91], and its degradation.

Furthermore, H_2_O_2_ exposure leads to protein oxidation, generating carbonyl groups, protein phosphorylation, and phospholipid de-esterification, which are detectable through FTIR spectra [92, 93]. A slight H_2_O_2_ concentration-dependent effect was observed, characterized by increased intensity of the phosphate bands at 1265 and 890 cm^− 1^, along with minor increases near 1715 cm^− 1^, likely attributable to the presence of carboxylic acid groups (Additional file 1: Fig. S7). This spectral pattern may be associated with protein phosphorylation and lipid peroxidation, leading to the release of free fatty acids. The slow metabolism of *L. corymbifera* grown at suboptimal temperatures (lower than its optimal 35–40 °C), combined with limited carbon availability for energy production, likely promotes severe oxidative protein damage. These damaged proteins appeared to accumulate as they were not efficiently removed by the fungus’ internal recycling mechanisms, ultimately leading to the phenomenon described here.

## Conclusion

Sublethal H₂O₂ exposure affected biomass production only at concentrations near the respective MIC values (1–19 mM). These effects included oxidative damage to proteins in *L. corymbifera* and minor reductions in lipid and polyphosphate accumulation in *R. stolonifer* and *M. circinelloides*. However, at concentrations typically used to enhance lignocellulose saccharification by boosting LPMO activity (1-240 µM), all the strains tolerated H_2_O_2_ without detectable sublethal effects on biochemical composition. We conclude that these strains are compatible with H_2_O_2_-enhanced SSF processes, potentially improving their efficiency.

Notably, under the conditions evaluated in this study, H_2_O_2_ did not enhance lipid accumulation in any of the strains tested. This was neither the case for production of β-carotene in *M. circinelloides* in the present experimental set up (absence of light and H_2_O_2_ dose present from the start). However, it is possible that in other conditions, H_2_O_2_ could trigger the production of specific metabolites.

Lipogenesis was primarily driven by the C/N ratio and no significant effects, deleterious or beneficial, were found with addition of H_2_O_2_. Moreover, variation in C/N ratios during fermentation revealed diverse carbon allocation strategies among the Mucoromycota species, resulting in a new finding in the field. These differences were briefly discussed in terms of phylogenetic and ecological differences. In the follow-up study, the carbon diversion towards different metabolites will be analyzed and discussed in more detail.

The use of vibrational spectroscopy techniques (FTIR and FT-Raman) and the Duetz cultivation system enabled the rapid generation and analysis of a large dataset with minimal sample processing. Applying multivariate data analysis to spectral data allowed the detection of multiple effects on the biochemical fungal biomass composition associated with different conditions of C/N and H_2_O_2_ that otherwise would be overlooked using target analytics. Future studies could employ cultivations setups like flasks or bioreactors to further explore these findings under controlled conditions and in a quantitative approach.

## Supplementary Information

Below is the link to the electronic supplementary material.

Supplementary Material 1

## Data Availability

All datasets generated for this study are available in the Zenodo repository upon reasonable request from BZ: https://doi.org/10.5281/zenodo.16746917.

## Abbreviations

C/N: Carbon to nitrogen ratio
MFC: Minimal fungicidal concentration
MIC: Minimal inhibitory concentration
LPMOs: Lytic polysaccharide monooxygenases
SSF: Simultaneous saccharification and fermentation
FT: Fourier transformed
IR: Infrared
HTS: High throughput screening
MEA: Malt extract agar
PCA: Principal component analysis
EMSC: Extended multiplicative signal correction
SG: Savitzky-Golay filter
PC: Principal component
MC(VI): *Mucor circinelloides*
CB: *Cunninghamella blakesleeana*
RS: *Rhizopus stolonifer*
LC: *Lichtheimia corymbifera*
MA: *Mortierella alpina*
AG: *Absidia glauca*
UV: *Umbelopsis vinacea*
MH: *Mortierella hyalina*
