# Supplementary material for: Effect of hydrogen peroxide and carbon-to-nitrogen ratio on growth and biochemical profile in oleaginous mucoromycota
Source: Microb Cell Fact. 2025 Nov 12;24:232. doi: 10.1186/s12934-025-02863-1 (PMC12613774; doi:10.1186/s12934-025-02863-1)
Supplement: Supplementary file 1 — Supplementary Material 1 [file 12934_2025_2863_MOESM1_ESM.pdf]

**Supplementary information**

**Additional file 1**

# Effect of hydrogen peroxide and carbon-to-nitrogen ratio on growth and biochemical profile in oleaginous Mucoromycota

Cristian Bolaño Losada[1], Boris Zimmermann[1], Svein Jarle Horn[2], Achim Kohler[1], Volha Shapaval[1]

[1] Faculty of Science and Technology, Norwegian University of Life Sciences (NMBU), 1432 Ås, Norway

[2] Faculty of Chemistry, Biotechnology and Food Science, Norwegian University of Life Sciences (NMBU), 1432 Ås, Norway.

*Corresponding author Cristian Bolaño Losada cristian.bolano.losada@nmbu.no.

Table S1. Range of $H_2O_2$ sublethal concentrations evaluated for each fungal strain.

| **Species** | *Mucor circinelloides* | *Rhizopus stolonifer* | *Mucor circinelloides* | *Umbelopsis vinacea* | *Mortierella alpina* | *Mortierella hyalina* | *Absidia glauca* | *Cunningha-mella blackesleeana* | *Lichtheimia corymbifera* |
|---|---|---|---|---|---|---|---|---|---|
| **Collection no.** | **CCM F220** | **VKM F400** | **VI04473** | **CCM F539** | **ATCC 32222** | **VKM F1629** | **CCM F451** | **CCM F705** | **CCM F8077** |
| **Abbreviation** | **MC** | **RS** | **MCVI** | **UV** | **MA** | **MH** | **AG** | **CB** | **LC** |
| **$H_2O_2$ (mM)** | 0 | 0 | 0 | 0 | 0 | 0 | 0 | 0 | 0 |
| | 0.438 | 0.5 | 0.25 | 0.0625 | 0.0625 | 0.0625 | 0.125 | 0.25 | 0.25 |
| | 0.563 | 0.563 | 0.5 | 0.125 | 0.125 | 0.125 | 0.25 | 0.5 | 0.5 |
| | 0.875 | 1 | 1 | 0.25 | 0.25 | 0.25 | 0.5 | 1 | 1 |
| | 1.125 | 1.125 | 2 | 0.5 | 0.5 | 0.5 | 1 | 2 | 2 |
| | 1.75 | 2 | 4 | 1 | 1 | 1 | 2 | 4 | 4 |
| | 2.25 | 2.25 | 8 | 2 | 2 | | | | |
| | 3.5 | 4 | | | | | | | |
| | 4.5 | 4.5 | | | | | | | |
| | 7 | 8 | | | | | | | |
| | 9 | | | | | | | | |
| | 14 | | | | | | | | |

Table S2. Values of minimal fungicidal concentration (MFC) and minimal inhibitory concentration (MIC) for the set of Mucoromycota strains studied. n.a.: not available.

| **Strain** | **Collection number** | **MFC ($H_2O_2$ mM)** | **MIC ($H_2O_2$ mM)** |
|---|---|---|---|
| *Mucor circinelloides* | CCM F220 | 25 | 18.7 |
| *Mucor circinelloides* | VI04473 | 12.5 | 9.38 |
| *Cunninghamella blakesleeana* | CCM F705 | 9.38 | 9.38 |
| *Rhizopus stolonifer* | VKM F400 | 9.38 | 9.38 |
| *Lichtheimia corymbifera* | CCM F8077 | 9.38 | 4.69 |
| *Mortierella alpina* | ATCC 32222 | n.a. | 4 |
| *Absidia glauca* | CCM F451 | 3.13 | 3.13 |
| *Umbelopsis vinacea* | CCM F539 | 2.34 | 1.56 |
| *Mortierella hyalina* | VKM F1629 | 1.17 | 1.17 |

* MFC calculation it is based in the concentration that kills 99.9% of the initial number of spores used while the MIC assay for *M. alpina* was based on mycelium. Therefore, MFC from *M. alpina* was technically impossible to define in such terms.

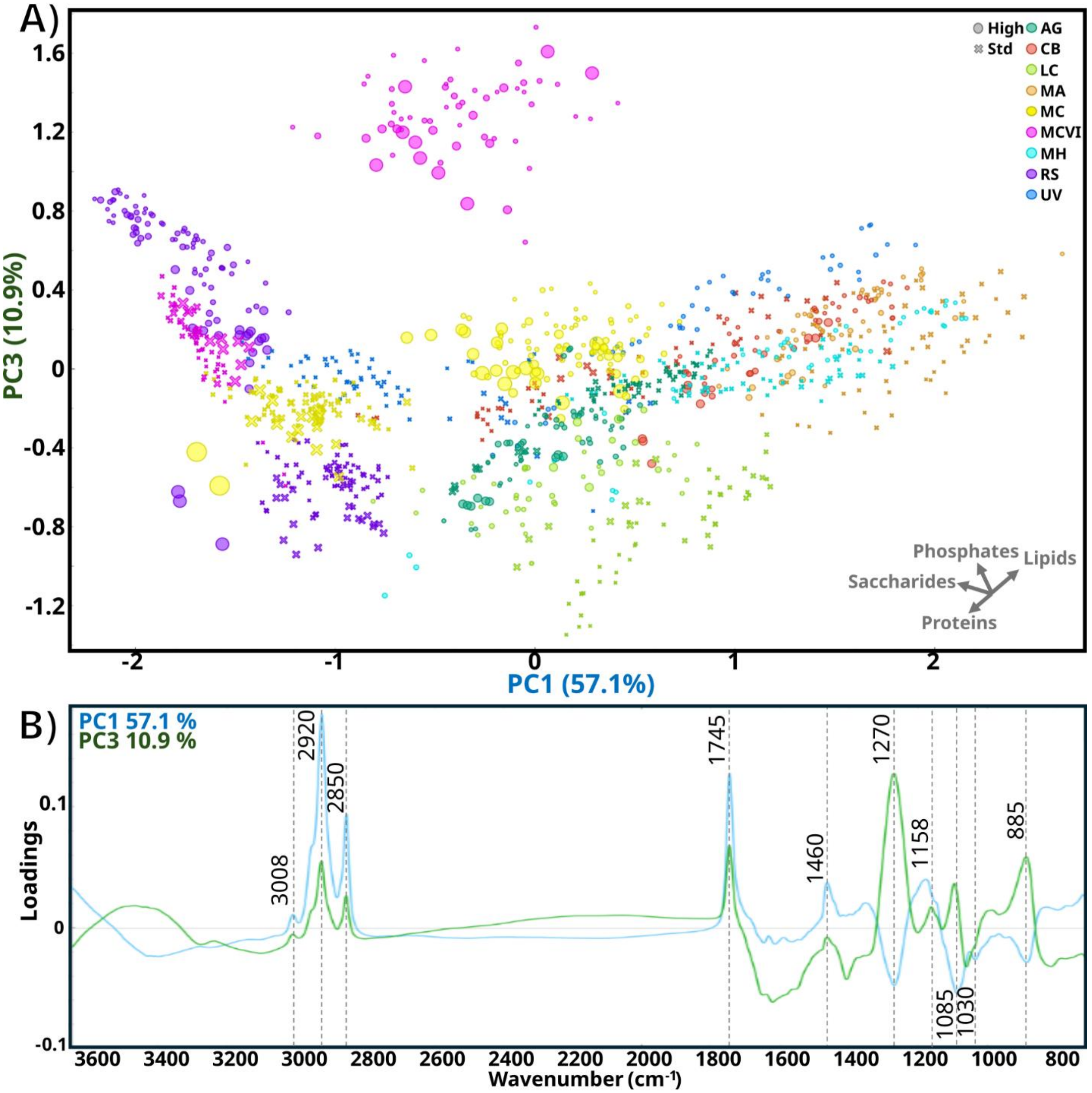


Figure S1. PCA score and loadings plots of FT-IR spectra of *A. glauca* (AG), *C. blakesleeana* (CB), *L. corymbifera* (LC), *M. alpina* (MA), *M. circinelloides* (MC and MCVI strains), *M. hyalina* (MH), *R. stolonifer* (RS), and *U. vinacea* (UV) grown at standard (✖) and high (●) C/N and different concentrations of $H_2O_2$ represented by relative proportional symbol size (larger symbol size corresponds to higher concentration). A) Score plot using PC1 and PC3 with an explained variance of 57.1% and 10.9% respectively. A vector axes are provided as approximate indications of the directions of the relative increase of each metabolite. Color transparent dash-line arrows indicate the progression of each species from standard to high C/N conditions. B) Loadings plot of PC1 and PC3 with the most relevant peaks assigned. The explained variance for the first five PC was: 57.1%, 16.5%, 10.9%, 4.7%, and 3%.

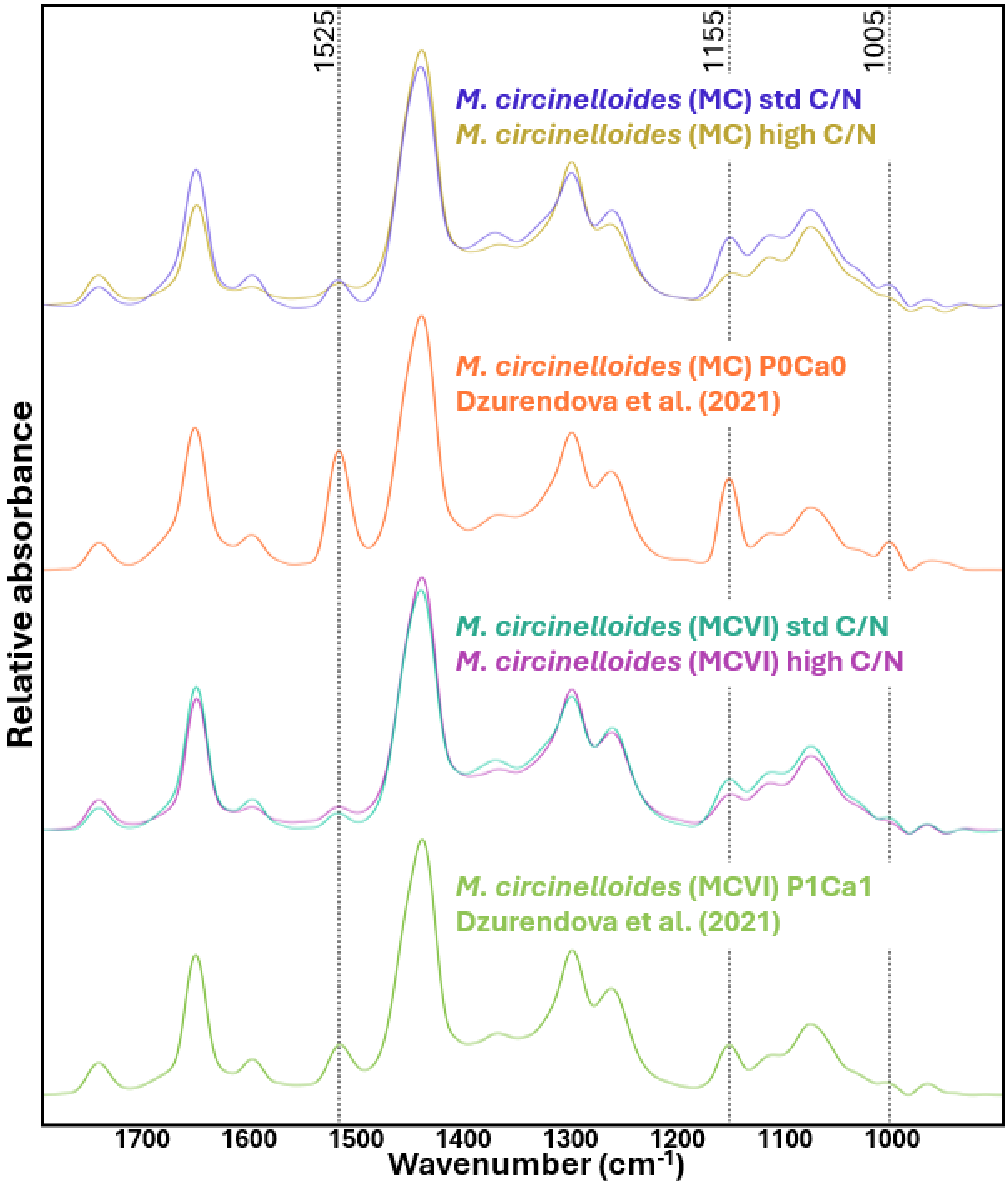


Figure S2. Comparison of FT-Raman spectra from *Mucor circinelloides* strains (MC and MCVI) grow at standard and high C/N medium and FT-Raman spectra of the same strains from Dzurendova et al. (2021) chosen at specific conditions (P: phosphate level, Ca: calcium level), which contained higher amounts of carotene than in the present study. Wavenumbers 1525, 1155 and 1005 $cm^{-1}$ are marked to indicate the presence of carotenes in the samples.

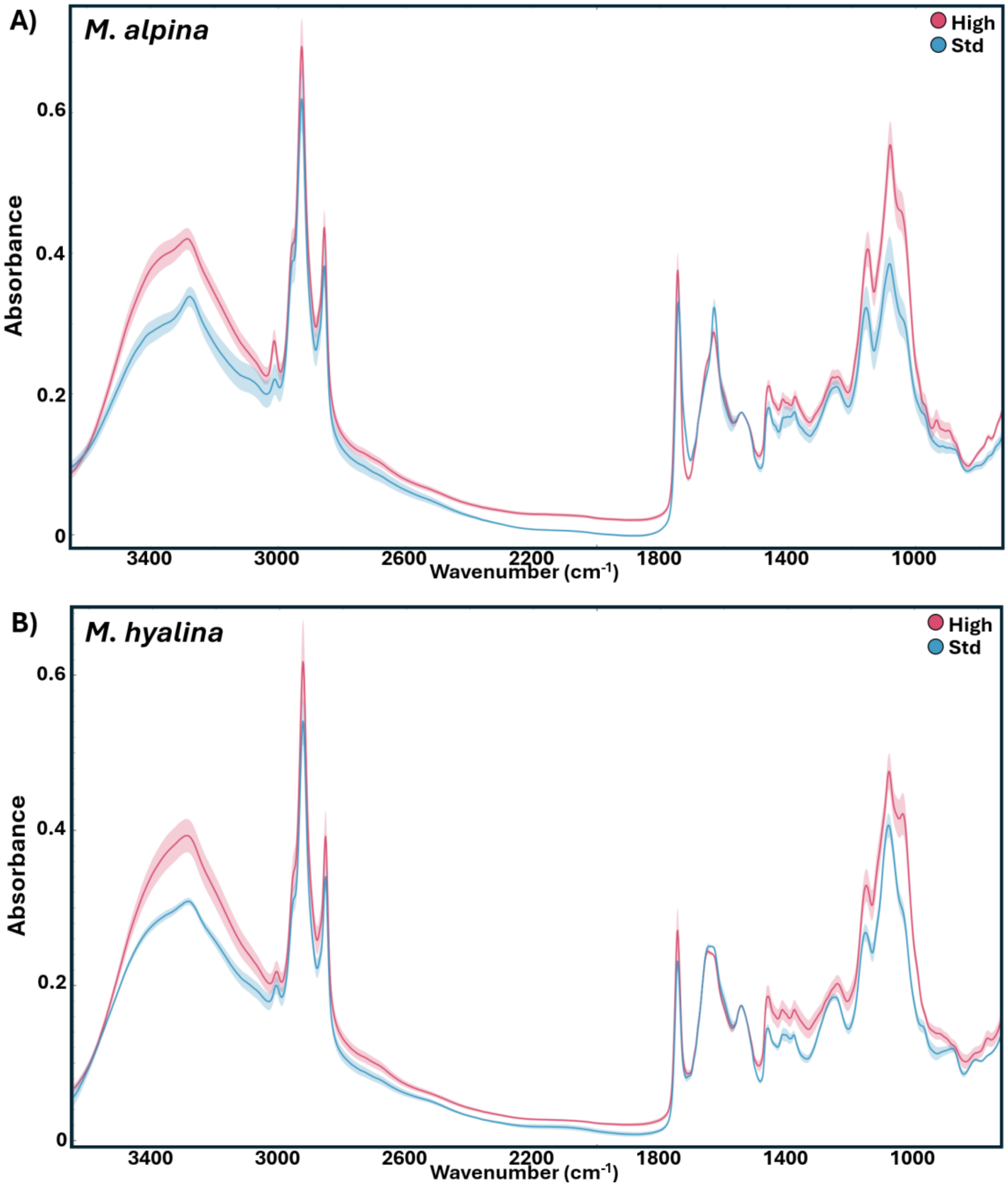


Figure S3. A) Spectra normalized by amide II from A) *Mortierella alpina* (MA) and B) *Mortierella hyalina* (MH) grown at standard and high C/N without $H_2O_2$.

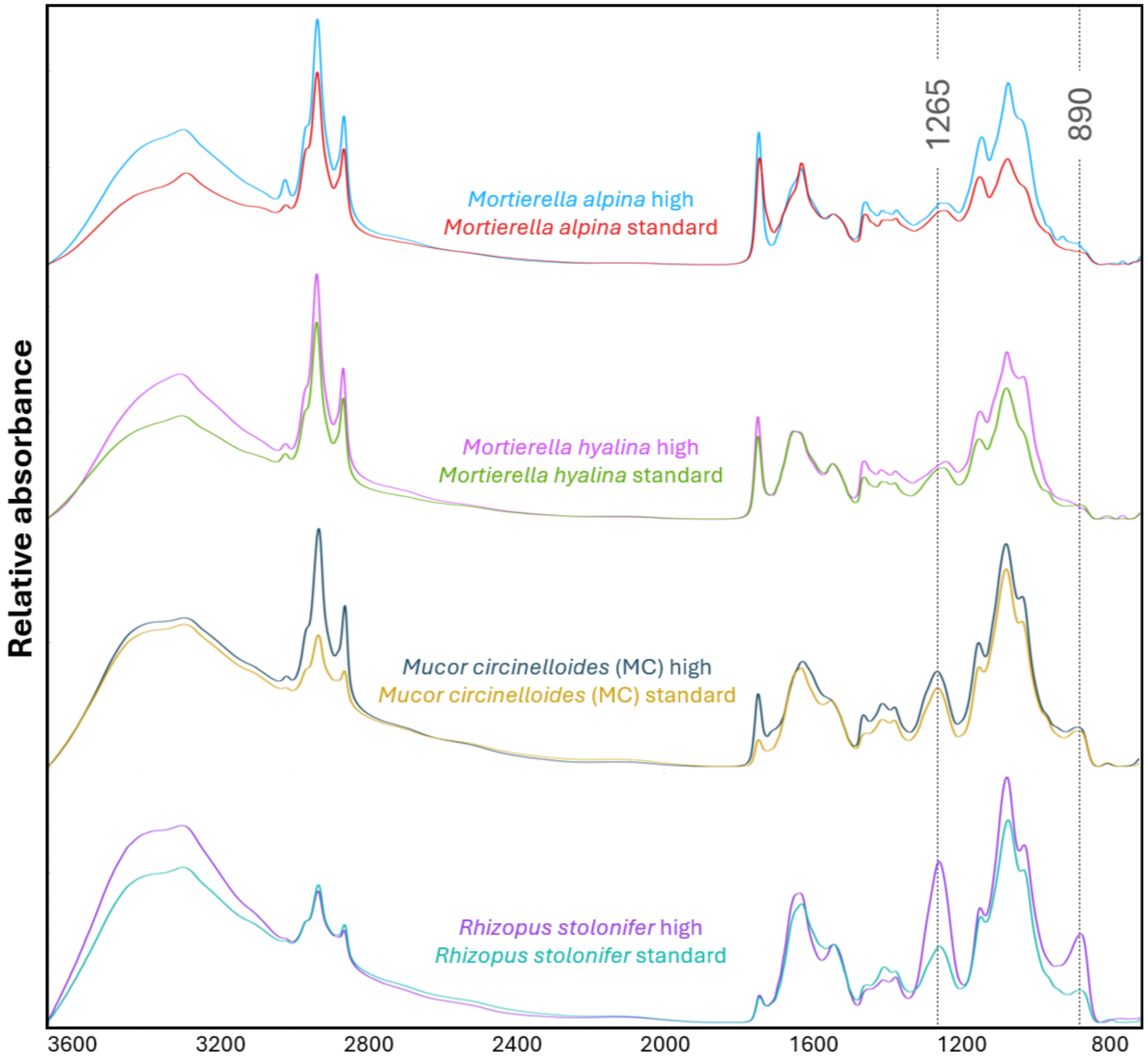


Figure S4. Comparison of phosphate-associated peaks (1265 $cm^{-1}$ and 890 $cm^{-1}$) in FTIR-HTS spectra from *M. alpina*, *M. hyalina*, *M. circinelloides* (MC), and *R. stolonifer* grown at standard and high C/N media without $H_2O_2$. Spectra were preprocessed by rubber band baseline correction and normalized by amide II.

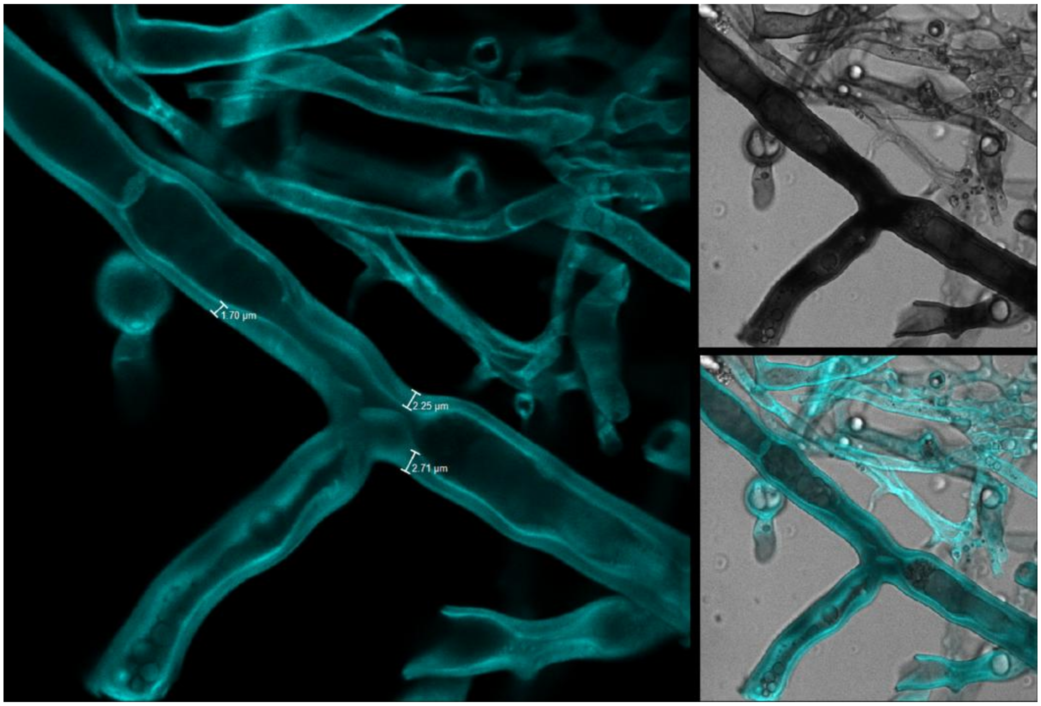


Figure S5. Pictograms of *Cunninghamella blakesleeana* stained with calcofluor white stain observed with confocal microscopy. View of fluorescence and brightfield channel separately and merged. The thickness of cell walls is marked with white bars and the corresponding value (1.7-2.7 µm).

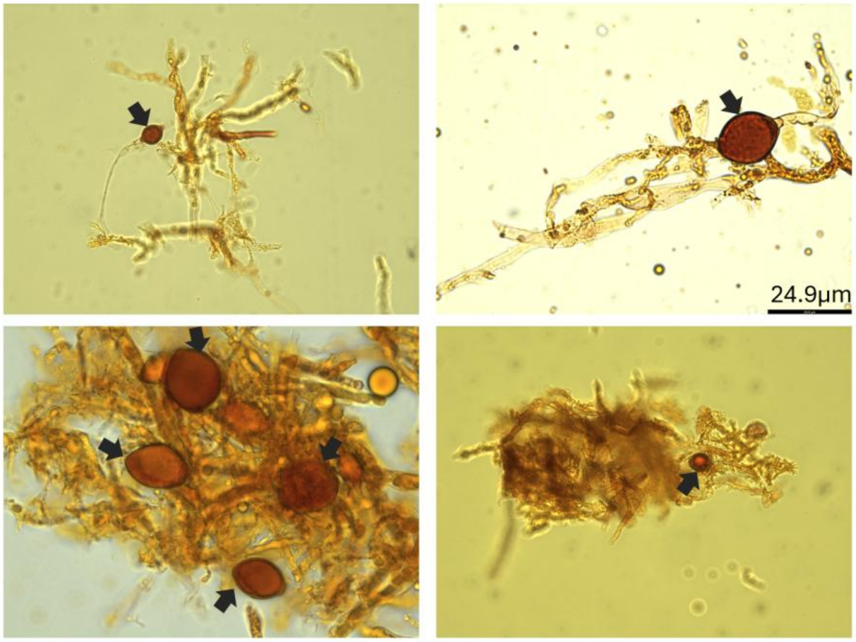


Figure S6. Pictograms of *Mortierella hyalina* grown in standard C/N medium stained with Lugol's reagent. Black arrows were included to indicate the presence of chlamydospores. The dark brown coloration produced by the staining indicates high content of glycogen and/or lipids.

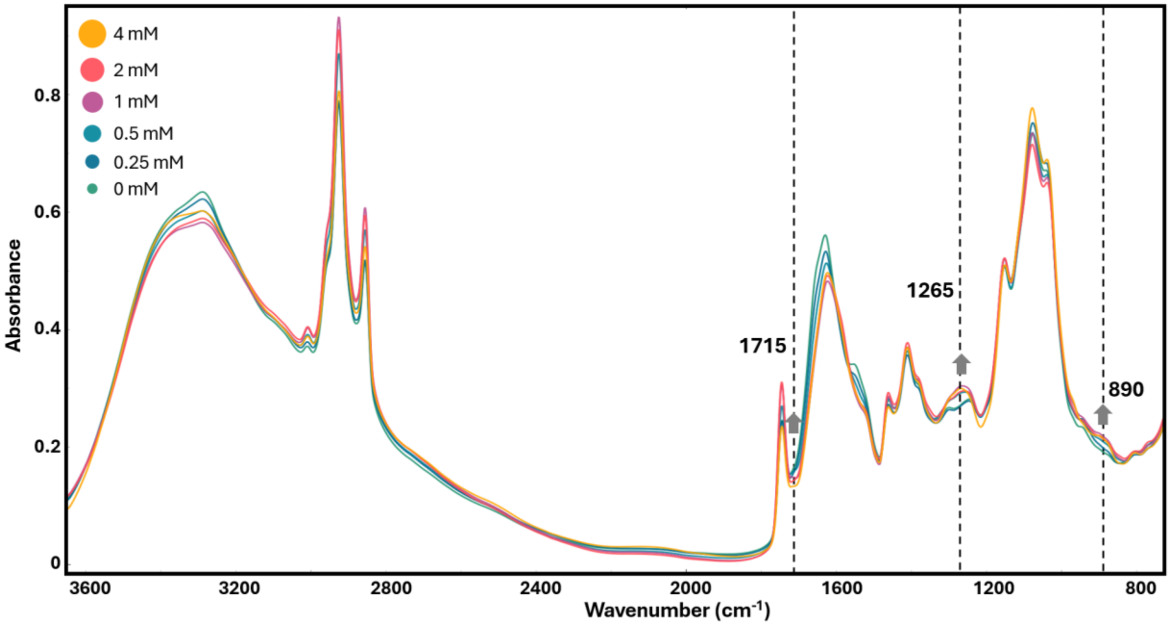


Figure S7. EMSC corrected spectra of *L. corymbifera* grown in standard C/N medium under different concentrations of $H_2O_2$. Dash lines and grey arrows indicate small increases in specific wavenumbers that can be associated with protein phosphorylation and lipid peroxidation.
